# Suppressive activity of Vδ2^+^ γδ T cells on αβ T cells is licensed by TCR signaling and correlates with signal strength

**DOI:** 10.1007/s00262-019-02469-8

**Published:** 2020-01-25

**Authors:** Karin Schilbach, Naomi Krickeberg, Carlotta Kaißer, Simon Mingram, Janika Kind, Gabrielle M. Siegers, Hisayoshi Hashimoto

**Affiliations:** 1 Department of Pediatric Hematology and Oncology, University Children’s Hospital Tübingen, Hoppe-Seyler Street 1, 72076 Tübingen, Germany; 2 Department of Oncology, University of Alberta, Edmonton, AB Canada

**Keywords:** γδ T cells, TCR signal strength, IPP, Immunosuppression, TCR-induced immune suppression

## Abstract

Despite recent progress in the understanding of γδ T cells’ roles and functions, their interaction with αβ T cells still remains to be elucidated. In this study, we sought to clarify what precisely endows peripheral Vδ2^+^ T cells with immunosuppressive function on autologous αβ T cells. We found that negatively freshly isolated Vδ2^+^ T cells do not exhibit suppressive behavior, even after stimulation with IL-12/IL-18/IL-15 or the sheer contact with butyrophilin-3A1-expressing tumor cell lines (U251 or SK-Mel-28). On the other hand, Vδ2^+^ T cells positively isolated through TCR crosslinking or after prolonged stimulation with isopentenyl pyrophosphate (IPP) mediate strong inhibitory effects on αβ T cell proliferation. Stimulation with IPP in the presence of IL-15 induces the most robust suppressive phenotype of Vδ2^+^ T cells. This indicates that Vδ2^+^ T cells’ suppressive activity is dependent on a TCR signal and that the degree of suppression correlates with its strength. Vδ2^+^ T cell immunosuppression does not correlate with their Foxp3 expression but rather with their PD-L1 protein expression, evidenced by the massive reduction of suppressive activity when using a blocking antibody. In conclusion, pharmacologic stimulation of Vδ2^+^ T cells via the Vδ2 TCR for activation and expansion induces Vδ2^+^ T cells' potent killer activity while simultaneously licensing them to suppress αβ T cell responses. Taken together, the study is a further step to understand—in more detail—the suppressive activity of Vδ2^+^ γδ T cells.

**Electronic supplementary material:**

The online version of this article (10.1007/s00262-019-02469-8) contains supplementary material, which is available to authorized users.

## Introduction

T lymphocytes are divided into two subsets by their expression of T cell antigen receptors (TCRs): αβ T cells (combination of an α chain and a β chain) and γδ T cells (combination of a γ chain and a δ chain). γδ T cells constitute a major T cell population in the epithelial tissues but represent a rare (typically 1–15%) T cell population in the peripheral circulation with the majority (50–95%) of γδ T cells carrying a Vγ9Vδ2 TCR [1]. A unique and specific feature of human Vγ9Vδ2 T cells (Vδ2^+^ T cells) is their TCR-dependent recognition of phosphoantigens (PAgs), metabolites of the phosphorylated isoprenoid pathway. Because of γδ T cells’ distinctive features—potent antitumor effect and independence from MHC restriction—a special interest has been taken in the application of Vδ2^+^ T cells in cancer immunotherapy [2, 3]. However, phase II trials for evaluating the efficacy of adoptive transfer and in vivo expansions of Vδ2^+^ T cells have resulted in limited clinical response in solid tumors thus far, even though many clinically unresponsive patients exhibited sustained Vδ2^+^ T-cell activation and proliferation [4–13]. Therefore, the mechanism which prevents Vδ2^+^ T cells from eliciting long-lasting antitumor effects in vivo needs to be elucidated. γδ T cells are known to inhibit or suppress the maturation and/or activation of other immune cells under certain conditions [14, 15]. In particular, their interaction with effector αβ T cells is of great interest with respect to a potential regulatory function of γδ T cells in cancer [16–18].

A tumor-infiltrating immunosuppressive Vδ2^+^ T cell population has been identified in multiple types of solid cancers [19, 20]; an in vitro study suggested that tumor-infiltrating γδ T cells inhibit αβ T cell activation via PD-1/PD-L1 ligation [19]. Yet, consensus has not been reached on whether or under which condition(s) Vδ2^+^ T cells exert suppressive function on αβ T cells. Casetti et al*.* showed that up to 30% of Vδ2^+^ T cells express the Foxp3 transcription factor when they are activated by isopentyl pyrophosphate (IPP) in the presence of IL-15 and TGF-β. Foxp3^+^-enriched Vδ2^+^ T cells, but not positively freshly isolated Vδ2^+^ T cells, displayed regulatory/immunosuppressive activity on αβ T cells when co-cultured with autologous PBMCs in the presence of anti-CD3/anti-CD28 mAb [21]. In the study of Peters et al*.* [22] neither IL-2 nor the combination of TGF-β1 and IL-15 induced regulatory functions in PAg-expanded γδ T cells on *Staphylococcus aureus* enterotoxin-stimulated CD4^+^CD25^-^ T cells. On the other hand, γδ T cells initially activated by anti-CD3/anti-CD28 in the presence of TGF-β and IL-15 suppressed CD4^+^CD25^-^ T cells although Foxp3 in γδ T cells was downregulated after transient expression. In contrast to Casetti’s paper, it was also reported that positively freshly isolated γδ T cells, which are Foxp3-negative, can potently suppress the in vitro proliferation of CD4^+^ T cells in the presence of anti-CD3/anti-CD28 mAb stimulation in the co-culture [22, 23]. In addition, Traxlmayr et al. [24] demonstrated that in the presence of antigen presenting cells, Vδ2^+^ T cells stimulated with IPP, but not negatively freshly isolated Vδ2^+^ T cells, can inhibit the proliferation of CD4^+^ and CD8^+^ αβ T cells reacting to strong recall antigens or allo-antigens. Combining these findings, Peters et al*.* [18, 22] suggested that γδ T cells exert their suppressive function only in the presence of anti-CD28 stimulation or antigen-presenting cells and that anti-CD28 stimulation rather than Foxp3 expression correlates closely with the suppressive capacity of γδ T cells. Moreover, as discussed by Wesch’s group, Foxp3 expression in suppressive human peripheral blood-derived Vδ2^+^ T cells cannot be detected with the Treg-specific 259D mAb [22] but can be identified with the PCH101 mAb that does not correlate with suppressive function [25, 26]. Clarity on this issue could be derived from methylation studies of the gene [27]. Taken together, it is still controversial as to whether Foxp3 expression is critical, or whether PAg stimulation is sufficient or additional cytokines are necessary for Vδ2^+^ T cells to exhibit cell-contact dependent suppression.

In the thymus, differences in signal strength dictate αβ versus γδ lineage choice through modulation of lineage specific transcription factors, while other signaling pathways that integrate with TCR signaling impact the resulting lineage outcome through altering activity of key proteins [28]. In light of this, it seemed likely that in the periphery, graded signals downstream of the TCR may result in differential functional maturation of T cell effector subpopulations while, at the same time, environmental cues such as cytokines might further modulate TCR signaling strength and effector function. The purpose of the present study therefore was to elucidate the role of the TCR in the acquisition of suppressive properties of peripheral human Vδ2^+^ T cells on autologous αβ T cells, specifically to address whether and how graded TCR stimulation and or cytokines control regulatory activities of Vδ2^+^ T cells. We examined the effect of proliferation inhibition and apoptosis induction mediated by negatively or positively freshly isolated Vδ2^+^ T cells obtained from healthy donors in comparison with those stimulated with IL-12/IL-18 (TCR bypass) + IL-15 and those after prolonged exposure to IPP with or without Th1 or Th2 cytokines. In addition, we tested the suppressive activity of Vδ2^+^ T cells in the presence or absence of a PD-1 blocking antibody. Next, to determine whether physiologic stimuli, such as the direct contact with tumor cells, affect the suppressive activity of Vδ2^+^ T cells, we exposed Vδ2^+^ T cells to a glioblastoma cell line (U251) or a melanoma cell line (SK-Mel-28) and subsequently examined these Vδ2^+^ T cells in mixed lymphocyte cultures (MLC) with anti-CD3/anti-CD28 stimulated autologous αβ T cells. Finally, we investigated the suppressive activity of Vδ2^+^ T cells in the presence or absence of CD28 stimulation. By employing Vδ2^+^ T cells that had experienced a range of stimuli, from none at all to supraphysiological levels, we identified conditions under which Vδ2^+^ T cells exerted suppressive effects on autologous αβ T cells.

## Methods

### Cell isolation and stimulation

PBMCs from healthy donors were isolated by Ficoll density gradient centrifugation (Biocoll Separating Solution; Biochrom AG, Germany). Vδ2^+^ T cells were isolated as follows: for positive isolation, PE-anti-Vδ2 antibody (clone B6, BD Pharmingen, Heidelberg, Germany), anti-PE Microbeads (Miltenyi Biotec, Bergisch Gladbach, Germany), and MS columns (Miltenyi Biotec) were used; and for negative isolation, initially pan γδ T cells were isolated by using a custom-made gammadelta T cell isolation kit (Stemcell Technologies, Vancouver, Canada), and Vδ1^+^ T cells were subsequently depleted by using PE-anti-Vδ1 mAb (clone REA173, Miltenyi Biotec), anti-PE MicroBeads, and LD columns (Miltenyi Biotec) according to the manufacturer’s protocol. CD3^+^ T cells were isolated through PE-anti-CD3 mAb (BD Pharmingen, clone HIT3α), anti-PE Microbeads (Miltenyi Biotec), and MS columns (Miltenyi Biotec) according to the manufacturer’s protocol. Purity of Vδ2^+^ T cells was > 98% for positive isolation and > 99% for negative isolation. Purity of CD3^+^ T cells was always > 99%. Stimulation with IPP: according to Casetti et al. [21], PBMCs were cultured for 10 days in a 24-well plate at a cell concentration of 4 × 10^6^ cells/ml in RPMI 1640 supplemented with 10% FBS, 2 mM l-glutamine (complete RPMI medium, all from Biochrom, Berlin, Germany), as well as IPP (20 μg/ml; Sigma Aldrich, Taufkirchen, Germany) and IL-2 (6.5 U/ml; Immuno Tools, Friesoythe, Germany) in the presence or absence of IL-15 (10 ng/ml; Immuno Tools), TGF-β1 (1.7 ng/ml, Immuno Tools) or IL-12 (10 ng/ml, Immuno Tools). On days 3, 6, and 9, half of the culture medium was replaced by fresh medium containing cytokines. TCR-bypass stimulation: negatively freshly isolated Vδ2^+^ cells were incubated for 24 h with IL-12 (50 ng/ml), IL-15 (50 ng/ml), and IL-18 (50 ng/ml, BioLegend, San Diego, CA, USA) in complete RPMI medium. Stimulation with anti-γδTCR mAb: culture plates were coated by overnight incubation with a TCRγδ mAb (clone IMMU510, BD Pharmingen, 1 μg/ml) at 4 ˚C. Then positively freshly isolated Vδ2^+^ cells were seeded in a flat bottom 96-well plate at 5×10^5^ cells/200 µl/well and cultured overnight. Viability of both freshly isolated and cultured Vδ2^+^ T cells was examined routinely via flow cytometry by using the live/dead fixable violet dead cell stain kit (Invitrogen, Carlsbad, CA, USA) and were always > 99%. We summarize denominations and corresponding isolation methods in Table 1.

**Table 1:** Denominations of Vδ2^+^ T cell entities and the stimulation/isolation procedure for their respective generation

Vδ2^+^ T cells	mode of isolation/generation
Vδ2^untouched^	Vδ2^+^ T cells negatively freshly isolated (untouched) from PBMCs
Vδ2^bypass^	Vδ2^+^ T cells negatively freshly isolated (untouched) from PBMCs, and stimulated with cytokines (IL-12/IL-18) [34–36], i.e., TCR bypass stimulation for 1 day
Vδ2^crosslink^	Vδ2^+^ T cells positively isolated from fresh PBMCs
Vδ2^IPP±cytokines^	Vδ2^+^ T cells stimulated with IPP within PBMCs for 10 days, thereafter positive isolation

### Mixed lymphocyte culture (MLC)

Isolated Vδ2^+^ T cells (1×10^5^ cells) were cultured at a 1:1 cell ratio in a flat bottom 96-well plate containing complete RPMI medium and IL-2 (50 U/ml) with autologous PBMCs. When untouched or TCR-bypass-stimulated Vδ2^+^ T cells were analyzed, autologous freshly isolated PBMCs had been activated by anti-CD3/anti-CD28 Dynabeads for 48 h beforehand (Thermo Fisher Scientific, Waltham, MA, USA). In this case, anti-CD3/anti-CD28 Dynabeads were removed before starting MLC to strictly avoid the unintentional TCR stimulation on Vδ2^+^ T cells. When using positively freshly isolated Vδ2^+^ T cells from blood or IPP-stimulated PBMCs, anti-CD3/anti-CD28 Dynabeads were added in MLC simultaneously when starting co-culture with autologous PBMCs without prior stimulation. To disrupt the interactions between PD-1 and PD-L1, we coated plates in advance overnight at 4 °C with a blocking antibody to PD-1 (Pembrolizumab) or isotype control at a concentration of 5 μg/ml in designated experiments. To investigate the role of CD28 stimulation on Vδ2^+^ T cells for their suppressive activity, IMMU510-stimulated Vδ2^+^ T cells were cultured at a 1:1 cell ratio with autologous PBMCs or T cells in a flat bottom 96-well plate coated with either anti-CD3 mAb (clone OKT3, BioLegend, 10 μg/ml) or with anti-CD3 in combination with anti-CD28 (clone 9.3, VWR, 2 μg/ml) mAb. Moreover, freshly isolated untouched Vδ2^+^ T cells were cultivated overnight in wells coated with anti-CD28 mAb (clone 9.3, 2 μg/ml), anti-TCRγδ mAb (IMMU510, 1 μg/ml), or both antibodies and then cultured at a 1:1 cell ratio with autologous T cells on anti-CD3 antibody-coated (clone OKT3, 10 μg/ml) wells in a flat bottom 96-well plate.

### Apoptosis assay

Cells were harvested and stained with PE-anti-Vδ2 (clone B6), APC-Cy7-anti-CD3 (clone SK7), both BD Bioscience, San Diego, USA; APC-anti-TCR αβ (clone BW242/412, Miltenyi Biotec), followed by FITC-annexin V and 7AAD (BD Pharmingen, via BD Biosciences, San Diego, USA) 24 h after MLC. Apoptotic cells were measured on a BD FACS CantoII flow cytometer and analyzed by using FlowJo Software v10 (Tree Star, Ashland, OR, USA). Apoptotic cells were defined as annexin V^+^ cells and relative frequency of apoptotic cells was calculated by subtracting the frequency of annexin V^+^ cells in CD3/CD28-stimulated αβ T cells in the absence of Vδ2^+^ T cells.

### Cell trace violet (CTV) proliferation assay

The proliferation of αβ T cells in MLC was examined using the CellTraceTM Violet Cell Proliferation Kit (Thermo Fisher Scientific). To this end autologous PBMCs were labelled with CTV before MLC. On day 3 of MLC, cells were stained with APC-Cy7-anti-CD3, APC-anti-TCR αβ, 7AAD and FITC-anti-TCR γδ (clone 11F2, BD Pharmingen) mAbs. Proliferation of αβ T cells was measured on a BD FACS CantoII flow cytometer and analyzed by using FlowJo Software. CTVlow cells were calculated as proliferating cells. Inhibition of proliferating cells was calculated by comparing with the frequency of CTVlow cells in CD3/CD28-stimulated αβ T cells in the absence of Vδ2^+^ T cells.

### Flow cytometric analysis

APC-anti-PD-1 (clone EH12.2H7), PE-Cy7-anti-PD-L1 (clone 29E2A3), APC-anti-CTLA4 (clone L3D10), APC-anti-perforin (clone B-D48), PE-anti-granzyme B (clone QA16A02, all from BioLegend), APC-Cy7-anti-CD3 (clone SK7), APC-anti-TCR αβ (clone BW242/412), 7AAD, FITC-anti-γδ TCR (clone 11F2), PE-anti-Vδ2 TCR antibody (clone B6), APC-H7-anti-CD28, FITC-anti-CD107a (clone H4A3, all from BD Pharmingen), and APC-anti-CD277 (clone BT3.1, Miltenyi Biotec) were used according to the manufacturer’s instructions. FITC-anti-CD107a antibody was added directly to the cell culture medium of Vδ2^+^ T cells with or without autologous PBMCs at 10 μl/ml and cells were incubated for 4 h at 37 °C with 10 μg/ml of brefeldin A (Biolegend) as well as 6 μg/ml of monensin (Golgi-Stop, BD). Rationale in brief: Monensin, which interacts with Golgi transmembrane transport, is the preferred choice when staining for only CD107a and not intracellular antibodies. Brefeldin A (BFA), which redistributes proteins from the Golgi to the ER, is commonly used when wanting to detect both CD107a and IFN. Both BFA and Monensin were used to detect CD107a and cytotoxins at the same time according to previous reports [29-31]. For intracellular cytokine staining of perforin and granzyme B, cells were fixed and permeabilized according to the manufacturer’s instructions (Nordic MUbio). Samples were measured on a BD FACS CantoII flow cytometer and analyzed using FlowJo Software v10.

### RNA extraction, cDNA synthesis

For transcript quantification, RNA was extracted with the use of RNeasy Mini Kit (Qiagen, Hilden, Germany) and reverse transcription was carried out using Superscript III First Strand Synthesis Super Mix (Life Technology, Germany) as described [32].

### Real-time PCR

For quantitative analysis, primers specific for Foxp3, PD-1, PD-L1, perforin, granzyme B, and GAPDH were used in real-time PCR with the SYBR Green kit (Promega, USA) in a BioRad C1000 Thermal cycler/CFX96 real-time System (BioRad, Germany). Briefly, 5 ng cDNA was added to a final volume of 10 µl/reaction containing 1 × SYBR Green PCR Master Mix (Promega, USA) and 100 nM of each primer. Thermal cycling conditions were: denaturation at 95˚C 2 min, 42 cycles: 95 °C/10 s, 59 °C/15 s and 72 °C/30 s for elongation. Primers: GAPDH: forward 5′-CCACATCGCTCAGACACCAT-3′ and reverse 5′-GGCAACAATATCCACTTTACCAGACT-3′. Foxp3: forward 5′-GAGAAGCTGAGTGCCATGCA-3′ and reverse 5′-GGAGCCCTTGTCGGATGAT-3′. PD-1: forward 5′-ATCAAAGAGAGCCTGCGGG-3′ and reverse 5′-GGTGGGCTGTGGGCACT-3′. PD-L1: forward 5′-AAATGGAACCTGGCGAAAGC-3′ and reverse 5′-GATGAGCCCCTCAGGCATTT-3′. Granzyme B: forward 5′- TTCGTGCTGACAGCTGCTCACT-3′ and reverse 5′- CTCTCCAGCTGCAGTAGCATGA-3′. Perforin: forward 5′-ACCAGCAATGTGCATGTGTCTG-3′ and reverse 5′- GCCCTCTTGAAGTCAGGGT-3’.

### Co-culture of Vδ2^+^ T cells with U251 glioblastoma or SK-Mel-28 melanoma cell lines

Adherent U251 (a glioblastoma cell line) or SK-Mel-28 (a melanoma cell line) cells were seeded in a 24-well plate at a cell concentration of 6 × 10^4^ cells/ml in complete RPMI medium on day 0. On day 1, 6 × 10^5^ isolated Vδ2^+^ T cells were added onto adherent tumor cells in a 24-well plate in complete RPMI medium supplemented with IL-2 (50 IU/ml). On day 3, Vδ2^+^ T cells were collected from the supernatant of culture medium for subsequent MLC.

### Cytotoxicity assay via electric cell substrates impedance sensing

The xCELLigence RTCA MP instrument (ACEA Biosciences) was utilized for cytotoxicity assays. First, 50 µl of culture media was added to each well of 96 well E-Plates (ACEA Biosciences) and the background impedance was measured. Dissociated adherent target cells (U251 or SK-Mel-28) were seeded at a density of 1 × 10^4^ cells/well of the E-Plate in a volume of 100 µl and allowed to passively adhere on the electrode surface. Post seeding, the E-Plate was transferred to the RTCA MP instrument inside a cell culture incubator and incubated for the first 24 h without effector cells. Then, γδ T cells—untreated or treated with zoledronate—were applied onto the adherent tumor cells, with different effector cell: target cell ratios (5:1, 2:1, 1:1, 0:1) in a volume of 100 µl. Changes in impedance were reported as Cell Index, which indicates attachment and adherence of cells to the plate’s electrode, every 30 min for the following 96 h as previously described [33]. Data analysis was performed using RTCA Software v1.2.1 (OLS).

### Statistical analysis

Statistical tests were performed with GraphPad PRISM v8. Unpaired and parametric data with same SDs can be assessed by Student’s *t* test. Comparisons between two groups were done using Student’s *t* test since both groups were normally distributed with equal variance. For comparisons among multiple groups, one-way ANOVA followed by Tukey’s multiple comparison test was used. The relationships between molecules expressed on γδ T cells and their suppressive activity were compared using Fisher’s exact test; the linear relationships were determined using Pearson's test. Statistical significance was defined as *p* < 0.05.

## Results

### Positively freshly isolated Vδ2^+^ T cells and Vδ2^+^ T cells stimulated by IPP in the presence or absence of relevant cytokines suppress the proliferation of autologous αβ T cells, while negatively isolated Vδ2^+^ T cells do not

To understand the role of the γδ TCR in determining functional differentiation into a regulatory phenotype, we examined peripheral Vδ2^+^ T cells after four different magnitudes of TCR stimulation, namely negatively freshly isolated Vδ2^+^ T cells (no stimulation), freshly untouched isolated Vδ2^+^ T cells with subsequent TCR-bypass stimulation (TCR-independent cytokine-mediated stimulation), fresh positively isolated Vδ2^+^ T cells (a single strong γδ TCR stimulus), and IPP-stimulated Vδ2^+^ T cells (strongest and continuous γδ TCR stimulation) for their suppressive activity by co-culturing them with anti-CD3/CD28 activated autologous αβ T cells. Vδ2^+^ T cells activated in a TCR-independent manner by inflammatory cytokines (TCR-bypass stimulation) [34–36] mimicked the mildest form of TCR stimulatory manipulation. In contrast, the anti-Vδ2 antibody crosslinks the TCR of Vδ2^+^ T cells during positive selection, resulting in one strong TCR stimulus. Activation by PAgs is currently the basis of two main strategies involving Vδ2^+^ T cells for cancer immunotherapy [37]: (1) in vivo administration of PAgs or aminobisphosphonates, and (2) adoptive transfer of ex vivo expanded Vδ2^+^ T cells by PAgs. Therefore, it is of critical importance to know whether PAg-stimulated Vδ2^+^ T cells have suppressive function or not. Thus γδ T cells stimulated for 10 days with IPP and subsequent positive isolation via the TCR using MACS technology represented the maximum TCR stimulatory manipulation in our model system. Since IL-15 and IL-12 alone or in combination enhanced the activation of PAg-stimulated γδ T cells [24, 36, 38] and TGF-β/IL-15 induced Foxp3 in Vδ2^+^ T cells, which then suppressed the proliferation of αβ T cells [21], TCR stimulation—where indicated—was combined with either one of these cytokines or combinations thereof.

**Fig. 1:**
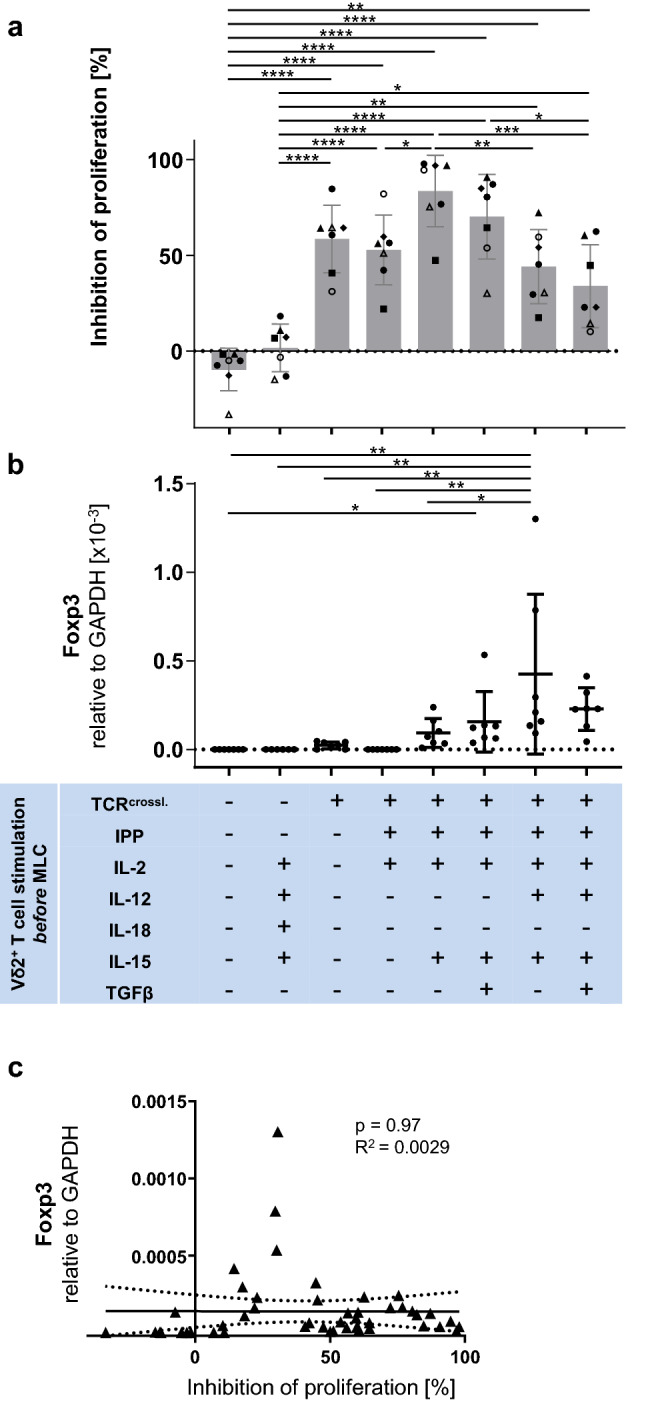
Impact of differentially stimulated γδ T cells on the proliferation of autologous αβ T cells. Isolated Vδ2^+^ T cells after indicated stimulations were washed and cultured at a 1:1 cell ratio with autologous CTV-labelled PBMCs activated by anti-CD3/anti-CD28 Dynabeads. **a** Inhibition of αβ T cell proliferation by autologous Vδ2^+^ T cells. On day3 of MLC, proliferation of αβ T cells was measured with flow cytometry and inhibition of proliferating cells was calculated. Each symbol indicates an individual donor culture. The data were generated using Vδ2^+^ T cells obtained from seven different healthy donors. **b** qPCR of Foxp3 expression in Vδ2^+^ T cells. Vδ2^+^ T cells were analyzed by qPCR. The data were generated using Vδ2^+^ γδ T cells obtained from seven different donors. One-way ANOVA followed by Tukey’s multiple comparison test was used. Bars represent the mean ± SD. *p  < 0.05, **p < 0.01, ***p < 0.001, ****p < 0.0001. **c** Correlation between mRNA levels of Foxp3 and proliferation inhibition. The relationships between Foxp3 expression on Vδ2^+^ T cells and their inhibitory activity were compared using Fisher’s exact test; the linear relationships were determined using Pearson’s test. TCR^crossl.^: Positive isolation of Vδ2^+^ T cells through anti-Vδ2 antibody.

Untouched Vδ2^+^ T cells did not exhibit any inhibitory effect on the proliferation of anti-CD3/anti-CD28 stimulated αβ T cells, while TCR-bypass stimulation with IL-12/IL-18 [39] combined with IL-15 resulted in slight effects, this constituting only 16% of the inhibition mediated by TCR-crosslink-stimulated cells (average 68% suppression, Fig. 1a). Fresh Vδ2^+^ T cells positively isolated through an anti-TCR Vδ2 antibody, however, significantly suppressed the proliferation of autologous αβ T cells (Fig. 1a). IPP stimulation was performed in diverse cytokine contexts. We investigated the suppressive activity of IPP-stimulated, IPP/IL-15-stimulated, IPP/IL-15/TGF-β-stimulated, IPP/IL-15/IL-12-stimulated, and IPP/IL-15/TGF-β/IL-12-stimulated Vδ2^+^ T cells. Vδ2^+^ T cells of all these cohorts significantly suppressed the proliferation of anti-CD3/anti-CD28-stimulated αβ T cells (Fig. 1a). Among them, IPP/IL-15-stimulated Vδ2^+^ T cells exhibited the strongest inhibitory effect on proliferation. IPP/IL-15/IL-12-stimulated cells exhibited a weaker inhibitory effect on proliferation than IPP/IL-15-stimulated cells, and IPP/IL-15/IL-12/TGF-β-stimulated cells showed weaker inhibitory effects on proliferation than IPP/IL-15/TGF-β-stimulated cells, suggesting that IL-12 in the presence of a strong TCR signal decreases this effect of Vδ2^+^ T cells. IPP/IL-15/TGF-β-stimulated cells exhibited weaker inhibitory effects on proliferation than IPP/IL-15-stimulated cells, and IPP/IL-12/IL-15/TGF-β-stimulated cells showed weaker inhibitory effects than IPP/IL-12/IL-15-stimulated cells, suggesting that TGF-β also alleviates the suppressive activity of IPP-stimulated Vδ2^+^ T cells in proliferation inhibition. This is in accordance with data from the literature that the signal transduction mechanism of IL-15 involves Lck and MAPK, thus synergizing with and enforcing the TCR signal pathway, enhancing TCR signal strength and thus suppressive function [40].

### Vδ2^+^ T cells stimulated by IPP with IL-15 or IL-12/IL-15 induce apoptosis of autologous αβ T cells

As shown in Supplementary Figure S1, compared to negatively freshly isolated Vδ2^+^ T cells, a significantly higher frequency of apoptotic αβ T cells was induced by positively selected IPP/IL-15/IL-12-stimulated Vδ2^+^ T cells or IPP/IL-15-stimulated Vδ2^+^ T cells. However, it is important to note that frequencies of annexin V^+^ apoptotic cells were always less than 10% among αβ T cells, rating the overall apoptosis induction effect as relatively small compared to the proliferation inhibiting effect shown in Fig. 1a. IPP/IL-15/IL-12-stimulated cells induced slightly more apoptotic αβ T cells than IPP/IL-15-stimulated cells, and IPP/IL-15/IL-12/TGF-β-stimulated cells showed slightly stronger apoptotic induction than IPP/IL-15/TGF-β-stimulated cells, suggesting IL-12 slightly increases apoptosis induction by Vδ2^+^ T cells. Addition of TGF-β to IPP/IL-15- or IPP/IL-15/IL-12-stimulated Vδ2^+^ T cells abrogated the apoptotic effect elicited by Vδ2^+^ T cells.

### IPP-stimulated Vδ2^+^ T cells express granzyme B and perforin at the protein level

To identify a potential correlation between suppression and apoptosis induction, we examined the expression of cytotoxic granules by Vδ2^+^ T cells, by first studying the mRNA expression of granzyme B and perforin in Vδ2^+^ T cells by qPCR (Supplementary Figure S2a). We found mRNA expression of granzyme B to be significantly higher on TCR-bypass-stimulated Vδ2^+^ T cells compared to untouched IL-2 stimulated Vδ2^+^ T cells; however, the reverse was true for perforin expression. In fact, TCR bypass stimulation induced significantly more granzyme B than any other condition tested. None of the positively selected Vδ2^+^ T cells expressed appreciable amounts of granzyme B or perforin mRNA. Accordingly, there was no significant correlation between mRNA levels of cytotoxic granules and apoptosis induction (Supplementary Figure S2b). Then we investigated the protein expression of granzyme B, perforin, and CD107a on Vδ2^+^ T cells by flow cytometry after expansion with PAgs before adding them into MLC (Supplementary Figure S3 left). During the process of cell killing, vesicles produced in an effector cell fuse with the target cell membrane, releasing cytotoxins such as perforin and granzymes. CD107a is a vesicle membrane protein that becomes transiently mobilized to the effector cell surface during this degranulation process [41]. The use of CD107a mobilization as a marker of degranulation has been described in NK, T, and also γδ T cells through flow cytometric analysis [42–45]. In contrast to mRNA levels, granzyme B, perforin, and CD107a protein expression was significantly upregulated after IPP/IL-15/IL-12 stimulation. Granzyme B and perforin expressions were also significantly upregulated by IPP/IL-15 but to a lesser extent compared to IPP/IL-15/IL-12 stimulation. In addition, we examined the same molecules before and after MLC and found CD107a expression upregulated and intracellular perforin expression downregulated on IPP/IL-15-stimulated Vδ2^+^ T cells after MLC with αβ T cells (Supplementary Figure S3, right), indicating degranulation of perforin upon cell contact with target cells. Interestingly, granzyme B expression remained unchanged. In conclusion, although only low levels of apoptosis are induced (below 10%), expression levels of granzyme B, perforin, and CD107a correlate exactly with these levels, suggesting apoptosis induction by Vδ2^+^ T cells is dependent on cytotoxic granules; however, this constitutes only a very small part of the overall regulatory activity of Vδ2^+^ T cells.

### Foxp3 expression on Vδ2^+^ T cells does not correlate significantly with their suppressive activity

In order to elucidate whether Foxp3 expression on Vδ2^+^ T cells is associated with their suppressive activity, we investigated the mRNA level of Foxp3 on Vδ2^+^ T cells. As shown previously [21], IPP-stimulated Vδ2^+^ T cells expressed higher levels of Foxp3 in the presence of TGF-β and IL-15 compared to most other conditions (Fig. 1b); however, IL-12/IL-15 also induced significantly higher expression of Foxp3 on IPP-stimulated Vδ2^+^ T cells compared to untouched Vδ2^+^ T cells or positively selected Vδ2^+^ T cells stimulated with IPP and IL-15 alone. No significant correlation was observed between Foxp3 expression and suppressive activity of Vδ2^+^ T cells (Fig. 1c).

### PD-L1 expression on Vδ2^+^ T cells correlates significantly with their suppressive activity

**Fig. 2:**
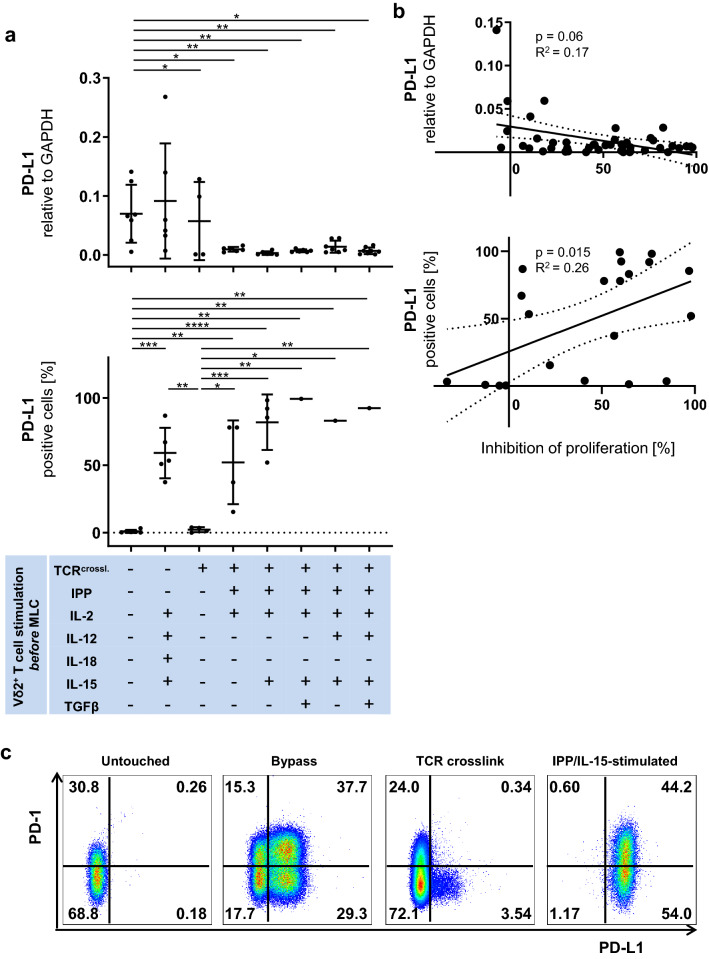
PD-L1 expression on Vδ2^+^ T cells correlates significantly with their suppressive activity. **a** (top) The mRNA expression of PD-L1 in Vδ2^+^ T cells. Vδ2^+^ T cells were analyzed by qPCR. **a** (bottom) The protein expression of PD-L1 on Vδ2^+^ T cells. Vδ2^+^ T cells were analyzed by flow cytometry. One-way ANOVA followed by Tukey’s multiple comparison test was used. Bars represent the mean ± SD. *p < 0.05, **p < 0.01, ***p < 0.001, ****p < 0.0001. **b** (top) Correlation between mRNA expression level of PD-L1 on Vδ2^+^ T cells and their suppressive activity. The relationships between mRNA level of PD-L1 expression on γδ T cells and their inhibition of proliferation were compared using Fisher’s exact test; the linear relationships were determined using Pearson’s test. **b **(bottom) Correlation between protein expression level of PD-L1 on Vδ2^+^ T cells and their suppressive activity. The relationships between PD-L1 protein expression on γδT cells and their proliferation inhibition activity were compared using Fisher's exact test; the linear relationships were determined using Pearson’s test. **c** Representative flow cytometric data of PD-L1 and PD-1 expression levels in Vδ2^+^ T cells. Representative flow cytometry plots are shown for representative untouched (Vδ2^untouched^), TCR-bypass-stimulated (Vδ2^bypass^), positively freshly isolated (Vδ2^crosslink^) and IPP/IL-15-stimulated Vδ2^+^ T cells (Vδ2^IPP/IL-15^). TCR^crossl.^: Positive isolation of Vδ2^+^ T cells through anti-Vδ2 antibody

**Fig. 3:**
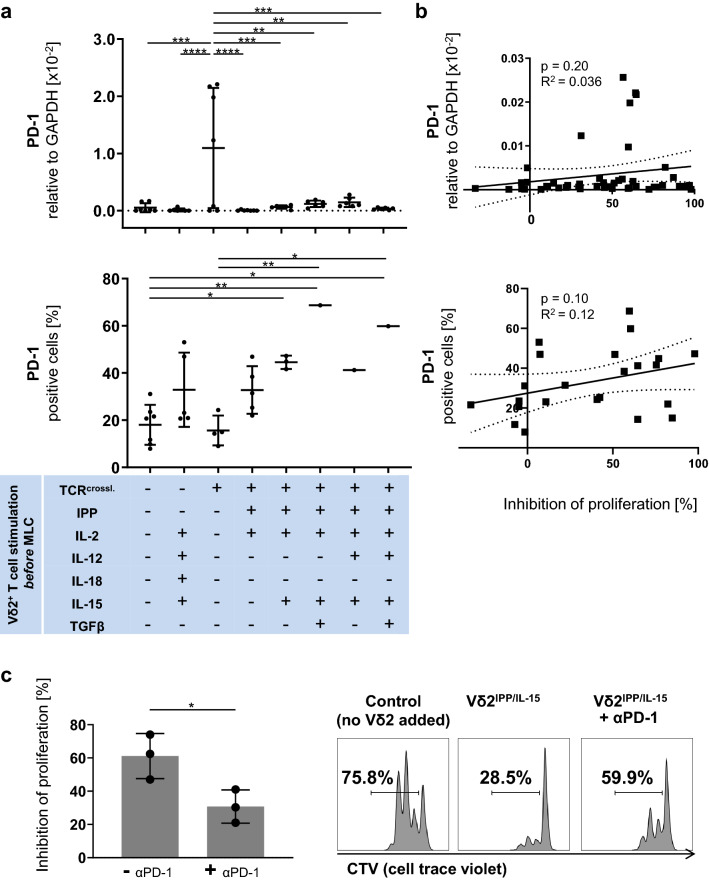
The suppressive activity of Vδ2^+^ T cells is PD-1/PD-L1-dependent. **a** (top) The mRNA expression of PD-1 in Vδ2^+^ T cells. Vδ2^+^ T cells were analyzed by qPCR. **a** (bottom) Protein expression of PD-1 on Vδ2^+^ T cells. Vδ2^+^ T cells were analyzed by flow cytometry. Since MLC was prioritized when only low cell numbers were available, data are available just from 1 donor for IPP/IL-15/TGF-ß, IPP/IL-15/IL-12 and IPP/IL-15/IL-12/TGF-ß stimulation. One-way ANOVA followed by Tukey’s multiple comparison test was used. Bars represent the mean ± SD. *p < 0.05, **p < 0.01, ***p < 0.001, ****p < 0.0001. **b** (top) Correlation between mRNA expression level of PD-1 on Vδ2^+^ T cells and their suppressive activity. The relationships between mRNA levels of PD-1 expression on γδ T cells and their inhibition of proliferation were compared using Fisher’s exact test; the linear relationships were determined using Pearson’s test. **b** (bottom) Correlation between protein expression level of PD-1 on Vδ2^+^ T cells and their suppressive activity. The relationships between PD-1 protein expression on γδ T cells and their proliferation inhibition activity were compared using Fisher’s exact test; the linear relationships were determined using Pearson’s test. **c** Inhibition of αβ T cell proliferation in αβ T cells by autologous Vδ2^+^ T cells in the presence of an anti-PD-1 antibody (Pembrolizumab). Vδ2^+^ T cells from three different donors were stimulated with IPP/IL-15 and were cultured at a 1:1 cell ratio with autologous CTV-labelled PBMCs activated by anti-CD3/anti-CD28 Dynabeads. PD-1/PD-L1 interaction in MLC was blocked by Pembrolizumab at a concentration of 5 μg/ml. Representative flow cytometric data of CTV in CD3/CD28-stimulated αβ T cells are shown from one donor together with their corresponding control (without co-culture with exogenous Vδ2^+^ T cells) on the right. Student’s t test was used to assess significance. Bars represent the mean ± SD. *p < 0.05. TCR^crossl.^: Positive isolation of Vδ2^+^ T cells through anti-Vδ2 antibody

Next, we investigated the mRNA levels of PD-L1 and PD-1 on Vδ2^+^ T cells by qPCR. We found that untouched Vδ2^+^ T cells expressed significantly higher mRNA levels of PD-L1 compared to all the other cohorts except TCR-bypass-stimulated Vδ2^+^ T cells (Fig. 2a, top). Unexpectedly, PD-L1 mRNA levels on Vδ2^+^ T cells did not correlate significantly with their suppressive activity (Fig. 2b, top). Since PD-1 and PD-L1 are regulated post-transcriptionally in immune cells [46, 47], we then investigated the protein expression of PD-1 and PD-L1 by flow cytometry. The reason why we could not analyze all the samples from all the donors is that since we prioritized MLC with autologous αβ T cells, isolated Vδ2^+^ T cells were numerically insufficient for flow cytometric analysis in some samples. Freshly isolated Vδ2^+^ T cells or positively freshly isolated Vδ2^+^ T cells did not express PD-L1, while its expression was significantly induced by the TCR-bypass stimulation or by IPP-stimulation (Fig. 2a, bottom). Protein levels of PD-L1 expression on Vδ2^+^ T cells significantly correlated with their suppressive activity (Fig. 2b, bottom); a representative example of flow cytometric analysis of Vδ2^+^ T cells from one donor is shown here (Fig. 2c). Positively freshly isolated Vδ2^+^ T cells expressed significantly higher mRNA levels of PD-1 compared to all the other cohorts (Fig. 3a, top). Protein levels of PD-1 were significantly upregulated by IPP/IL-15, IPP/IL-15/TGF-β, or IPP/IL-15/TGF- β/IL-12 compared to untouched Vδ2^+^ T cells (Fig. 3a, bottom). There was no significant correlation between the expression of PD-1 and the suppressive activity on Vδ2^+^ T cells (Fig. 3b).

### The suppressive activity of Vδ2^+^ T cells is PD-1/PD-L1-dependent

In order to reveal whether the suppressive activity of Vδ2^+^ T cells is dependent on the PD-1/PD-L1 axis, we blocked PD-1/PD-L1 interaction in MLC using immobilized anti-PD-1 antibody (Pembrolizumab) at a concentration of 5 μg/ml. While the apoptosis induction effect of IPP/IL-15- or IPP/IL-15/IL-12-stimulated Vδ2^+^ T cells was not altered (Supplementary Figure S4), the proliferation inhibition effect of IPP/IL-15-stimulated Vδ2^+^ T cells was abrogated by 50% by the anti-PD-1 antibody (Fig. 3c), indicating that the suppressive activity of Vδ2^+^ T cells is PD-1/PD-L1-dependent.

### Negatively freshly isolated Vδ2^+^ T cells do not suppress proliferation nor do they induce apoptosis of autologous αβ T cells after exposure to BTN3A1-expressing tumor cells alone

**Fig. 4:**
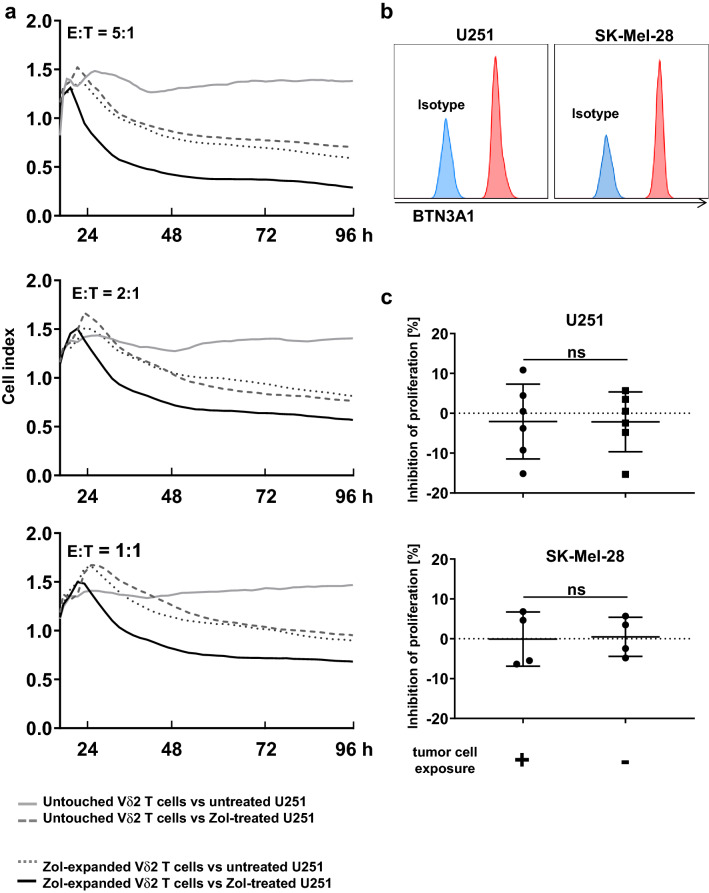
Exposure to BTN3A1-expressing tumor cells does not confer suppressive function to Vδ2^+^ T cells. **a** Anti-tumor activity of Vδ2^+^ T cells toward U251 cells. Negatively freshly isolated Vδ2^+^ T cells or zoledronate-stimulated Vδ2^+^ T cells were co-cultured with zoledronate-treated or untreated U251 cells (grown to 80% confluency) at different effector: tumor cell ratios (5:1, 2:1, 1:1, and 0:1 as control of tumor cell only). By using the Electric Cell-substrate Impedance Sensing, their cytotoxic effects were monitored. Anti-tumor activity of Vδ2^+^ γδ T cells from three healthy individuals was analyzed. Given are representative results for E: T ratios of 5:1, 2:1, and 1:1. **b** BTN3A1 expression on U251 and SK-Mel-28 cells. U251 or SK-Mel-28 cells were analyzed for expression of BTN3A1 together with an isotype control by flow cytometry. **c** Suppressive activity of Vδ2^+^ T cells on autologous αβ T cells after exposure to tumor cells. After co-culture with U251 or SK-Mel-28 cells, Vδ2^+^ T cells were harvested and subsequently cultured at a 1:1 cell ratio with autologous CTV-labelled PBMCs activated by anti-CD3/anti-CD28 Dynabeads. On day 3 of MLC, proliferation of αβ T cells was measured with flow cytometry and inhibition of proliferating cells was calculated. The data were obtained using Vδ2^+^ T cells from 6 different healthy donors for U251 and four different healthy donors for SK-Mel-28. Student’s *t* test was used. Bars represent the mean ± SD. *ns* not significant

The transmembrane butyrophilin (BTN) proteins are essential for human γδ T cell activation by PAgs [48]. As shown in Fig. 4a, while Vδ2^+^ T cells activated by zoledronate kill BTN3A1-expressing U251 cells through TCR stimulation, untouched freshly isolated Vδ2^+^ T cells do not. To associate physiologic relevance of anti-tumor activity and the suppressive effect of Vδ2^+^ T cells ex vivo, we first exposed untouched Vδ2^+^ T cells to BTN3A1-expressing tumor cells (U251 or SK-Mel-28, Fig. 4b) and consecutively co-cultured them with autologous αβ T cells. Exposure to the tumor cells alone in the absence of tumor lysis in vitro did not license the suppressive activity in Vδ2^+^ T cells in terms of apoptosis induction or proliferation inhibition of αβ T cells (Supplementary Figure S5 and Fig. 4c).

### CD28 stimulation is not essential for Vδ2^+^ T cell suppression of autologous αβ T cells

**Fig. 5:**
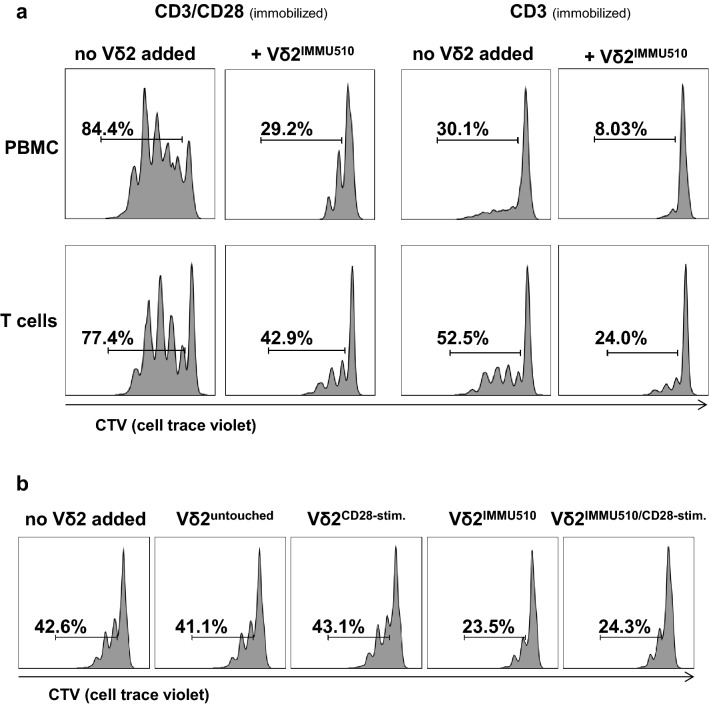
CD28 stimulation is not essential for Vδ2^+^ T cells to suppress autologous αβ T cells. **a** Suppressive activity of Vδ2^+^ T cells on autologous αβ T cells without CD28-stimulation. Vδ2^+^ T cells positively isolated from fresh peripheral blood were stimulated by immobilized anti-TCRγδ antibody (IMMU510, 1 μg/ml) and then co-cultured at a 1:1 cell ratio with autologous CTV-labelled autologous PBMCs or CD3^+^ T cells on CD3- or CD3/CD28-coated wells. On day 3 of MLC, proliferation of αβ T cells was measured with flow cytometry. Shown are the representative data from one donor. This experiment was repeated three times and always demonstrated the same trend. **b** Suppressive activity of Vδ2^+^ T cells with CD28 stimulation prior to MLC. Freshly isolated untouched Vδ2^+^ T cells were cultivated overnight in wells coated with anti-CD28 (clone 9.3, 2 μg/ml), or anti-TCRγδ antibody (IMMU510, 1 μg/ml), or both antibodies and then co-cultured with autologous T cells at a 1:1 cell ratio on anti-CD3 antibody-coated (OKT3, 10 μg/ml) wells. On day 3 of MLC, proliferation of αβ T cells was measured with flow cytometry. Shown are the representative data from one donor. This experiment was repeated three times and always demonstrated the same trend

Peters et al. suggested that CD28 stimulation correlates closely with the suppressive capacity of γδ T cells [18, 22], which is indeed plausible, since activated Vδ2^+^ T cells with high CD80/86 expression are potent antigen presenting cells to αβ T cells [49]. To determine whether CD28 stimulation has a role in the acquisition of a suppressive phenotype of Vδ2^+^ T cells, we first examined the suppressive activity of TCR-stimulated Vδ2^+^ T cells that were cultured in the absence of antigen presenting cells and CD28 stimulation prior to MLC (Fig. 5a). In order to avoid CD28 stimulation of Vδ2^+^ T cells from immune cells in PBMC, Vδ2^+^ T cells were not expanded by PAgs in PBMCs, but rather were freshly isolated from peripheral blood by positive selection (purity > 99%, Supplementary Figure S6) and stimulated with immobilized anti-TCRγδ antibody (IMMU510, 1 μg/ml) for 24 h, and only then co-cultured with autologous PBMCs or CD3^+^ T cells on anti-CD3- or anti-CD3/anti-CD28-coated wells. While anti-CD28 mAb can be either immobilized or added soluble, we chose immobilized anti-CD28 mAb for reasons of comparison with anti-CD3/anti-CD28 bead stimulation [50]. To omit CD28 stimulation from CD80/CD86-expressing cells in PBMCs, we co-cultured these Vδ2^+^ T cells—that had been positively selected and subsequently activated via immobilized IMMU510—with isolated CD3^+^ T cells, and compared these to those co-cultured with PBMCs (Fig. 5a). By comparing the proliferation inhibition effect by Vδ2^+^ T cells between CD3 stimulation and CD3/CD28 stimulation in MLC, we found that proliferation of αβ T cells was slightly more suppressed under sole CD3 stimulation than CD3/CD28 stimulation. This was the case regardless of which cells (autologous PBMCs or T cells) were used as targets, suggesting that CD28 stimulation may not be necessary for Vδ2^+^ T cells themselves to differentiate into a suppressive phenotype, yet CD28 stimulation may be relevant for the targets that get suppressed. Figure 5a depicts the representative data from one of three donor cultures. Data for donor 2 and 3 are provided in Supplementary Figure S7. Under CD3/CD28 stimulation (immobilized), TCR-stimulated Vδ2^+^ T cells inhibited the proliferation of αβ T cells in PBMCs by 65.4% and those in pure T cells by 44.6% (Fig. 5a). Under CD3 sole stimulation (immobilized), TCR-stimulated Vδ2^+^ T cells reduced the proliferation of αβ T cells in PBMCs by 73.3% and those in pure T cells by 54.3% (Fig. 5a). This experiment was repeated three times with different donors and always demonstrated the same trend (Fig. 5a and Supplementary Figure S7).

To further investigate the role of CD28 stimulation with respect to the induction of suppressive function of Vδ2^+^ T cells, we analyzed freshly isolated untouched Vδ2^+^ T cells (purity > 98%, contaminating cells were in the lymphocyte gate in forward versus side scatter (FSC vs SSC) and CD14 negative, thus unlikely monocytes, Supplementary Figure S8) cultivated in wells immobilized with anti-CD28, or anti-TCRγδ or both mAbs and then co-cultured them with autologous T cells stimulated with anti-CD3 mAb (OKT3). Different to in vivo situation where T cell proliferation is dependent on a second signal T cell proliferation solely by OKT3 was already proven in vitro in previous literatures in different settings [51–54]. Figure 5b illustrates the representative data from one of three donor cultures. Regardless of CD28 stimulation, untouched Vδ2^+^ T cells exerted no suppressive effect. Accordingly, TCRγδ-stimulated Vδ2^+^ T cells inhibited the proliferation of αβ T cells to a similar level in the absence or presence of CD28-stimulation (44.8% and 43.0%, respectively). These results indicate that CD28 stimulation does not induce additional suppressive activity on either untouched or TCR-stimulated Vδ2^+^ T cells.

## Discussion

Tumor-infiltrating γδ T cells have been considered the most favorable prognostic marker in many types of cancers [55]. While the adoptive transfer of ex vivo-expanded Vδ2^+^ T cells and even the adoptive transfer of allogeneic γδ T cells shows objective effects, complete remissions still remain anecdotical and the necessary repetitive in vivo activation shows limited responses [56]. The present study, therefore, examined γδ T cells’ suppressive behavior as one potential mechanism preventing Vδ2^+^ T cells from exerting long-lasting antitumor effects in vivo. Current in vitro and in vivo strategies employ pharmacological drugs targeting the Vδ2^+^ TCR for the induction of activation, proliferation, and tumor-lytic function of γδ T cells. By taking a closer look at the role of the TCR in this process, we demonstrate that the suppressive activity of Vδ2^+^ T cells on autologous αβ T cells correlates with the strength of γδ TCR signal and that the functional differentiation of γδ T cells into a suppressive phenotype is fine-tuned by the integration of signals arising from other environmental cues, such as cytokines. More specifically, negatively freshly isolated Vδ2^+^ T cells, TCR-bypass cytokine-stimulated negatively isolated Vδ2^+^ T cells, TCR-crosslinked freshly isolated Vδ2^+^ T cells, and IPP-stimulated TCR-crosslinked Vδ2^+^ T cells exhibit increasing inhibitory activities on αβ T cell proliferation, from the weakest to the strongest stimulation setting in this order. In contrast, direct contact with BTN3A1-expressing cells in vitro alone is not a sufficiently strong γδ TCR-stimulation to bestow suppressive activity on Vδ2^+^ T cells.

We examined peripheral Vδ2^+^ T cells with four different magnitudes of TCR stimulation. First, we showed that negatively freshly isolated Vδ2^+^ T cells (no stimulation) do not possess suppressive activity in accordance with Traxlmayr and colleagues’ findings [24]. While these authors used anti-CD4, CD16, TCRαβ cocktail antibodies for depleting unwanted cells, a custom-made kit for the isolation of untouched γδ T cells (omitting anti-CD16 antibody from the commercially available cocktail of antibodies) was used in the present study to avoid losing CD16^+^ Vδ2^+^ T cells because this fraction consists of 10–40% of peripheral Vδ2^+^ T cells in healthy donors [57]. In addition, we depleted Vδ1^+^ T cells to specifically analyze Vδ2^+^ T cells. Therefore, we believe that we studied the most purified (> 99%) untouched Vδ2^+^ T cells. Next, we found that TCR-bypass stimulation (TCR-independent cytokine-mediated stimulation) does not confer significant immunosuppressive function to negatively freshly isolated Vδ2^+^ T cells. To our knowledge, this is the first study to test the impact of TCR-bypass stimulation on the suppressive effect of truly untouched Vδ2^+^ T cells. Third, our study demonstrates that positively freshly isolated Vδ2^+^ T cells (a single γδ TCR stimulation) potently suppress the proliferation of co-cultured autologous αβ T cells that have been stimulated with anti-CD3/anti-CD28 mAb in whole PBMCs. Casetti et al. [21] concluded that positively isolated Vδ2^+^ T cells do not suppress the proliferation of anti-CD3/anti-CD28 mAb-stimulated PBMCs, while Peters et al. and Kuhl et al. showed that positively freshly isolated pan γδ T cells suppress the proliferation of CD4^+^ T cells in response to anti-CD3/anti-CD28 mAb stimulation [22, 23]. These conflicting results might be explained by the fact that Casetti et al. used not fresh PBMCs, but buffy coats and bead-sorted γδ T cells that had been frozen for use at later time points. Of utmost importance, however, is our new finding that crosslinking the γδ TCR merely once leads to a remarkable suppressive function of Vδ2^+^ T cells. Finally, we reveal that IPP-stimulated Vδ2^+^ T cells (strongest and continuous γδ TCR stimulation) exhibit the most robust suppressive activity. Intriguingly, untouched Vδ2^+^ T cells do not suppress autologous αβ T cells even after exposure to BTN3A1^+^ tumor cells. As tumor-infiltrating γδ T cells are thought to be the most favorable prognostic marker in many types of cancers [55], this finding suggests that tumor cells do not license suppressive functions of γδ T cells. On the other hand, in vivo or ex vivo stimulation of Vδ2^+^ T cells via pharmacologic drugs for activation and expansion may dampen the anti-tumor reactivity of αβ T cells indirectly by reducing their number, which points to a thus far unappreciated limitation to adoptive Vδ2^+^ T cell therapy for cancer patients. However, since T cells with high capacity of proliferation and those with high potential of cytokine-production or anti-tumor cytolytic function are different populations, we cannot conclude whether γδ T cells dampen αβ T cell responses per se. They may actually enhance the cytolytic potential of suppressed αβ T cells, which we did not examine in this study.

The impact of cytokines on γδ T cells, especially on the suppressive function of γδ T cells, is largely unknown. In this study, we show that IL-12 in the presence of a strong TCR signal decreases the inhibitory effect on αβ T cell proliferation by Vδ2^+^ T cells yet very slightly increases apoptosis induction by Vδ2^+^ T cells (Fig. 1a and Supplementary Figure S1). An interesting study by Traxlmayr et al. [24] showed that IL-12 enhanced the suppressive activity of γδ T cells against memory T cells, stimulated by recall antigens, but not against naïve T cells, responding to neoantigens. Combined together, the modulation of the features of Vδ2^+^ T cells by IL-12 is still controversial and warrants further investigation. In addition, as shown in Fig. 1a and Supplementary Figure S1, MLC data suggest that TGF-β alleviates the suppressive activity of IPP-stimulated Vδ2^+^ T cells both in proliferation inhibition and apoptosis induction. From the literature we know that TGF-β suppresses the effector function of γδ T cells [58–60] and inhibits IL-12-induced proliferation and activation of T cells [61]; therefore, it is likely that IL-12 and TGF-β also counter-regulate and cancel out the TCR-mediated activation status of Vδ2^+^ T cells in the current study. Moreover, we show that IL-15 enhances the suppressive activity of IPP-stimulated Vδ2^+^ T cells both via inhibiting proliferation and inducing apoptosis. This finding is in accordance with a previous report demonstrating that the addition of IL-15 to γδ T cell cultures results in a more activated phenotype, a higher proliferative capacity, and increased cytotoxicity of γδ T cells [38]. Most noteworthy, however, is that IL-15 signaling involves Lck and MAPK in its downstream cascade [40], mimics T cell receptor crosslinking in the induction of gene expression, and shares overall downstream events with anti-CD3 stimulation in memory T cells, thus is fortifying TCR signaling [62].

Immunotherapeutic strategies that aim to activate and expand Vδ2^+^ T cells in vitro or in vivo stimulate Vδ2^+^ γδ T cells via their TCR to maximize their cytotoxic properties, dependent on cytotoxic mediators. Due to the observed dichotomy in Vδ2^+^ T cells, i.e., that a TCR signal induces both anti-tumoral as well as a suppressive phenotype, we investigated the role of apoptosis induction in the regulatory function of Vδ2^+^ T cells. Consistent with results shown by Peters et al., we found the contribution of apoptosis marginal compared to that of proliferation inhibition (Fig. 1a and Supplementary Figure S1). Peters et al. examined the relevance of Fas/FasL and TRAIL/TRAILR in suppressive assays simultaneously by using blocking antibodies for these apoptosis cascades and did not detect any effect on overall suppressive activity [22].

Thus, although we and others have identified an array of conditions that create a suppressive phenotype in Vδ2^+^ γδ T cells, the molecules involved still remain largely unknown. While extensive research has convincingly established the key contribution of costimulation on αβ T cell functions, the functional relevance of co-stimulatory/co-inhibitory molecules on γδ T cells in the context of suppressive activity as well as a role for CD28 in the acquisition of a suppressive phenotype of Vδ2^+^ γδ T cells remains to be defined. CD28 is expressed by 40–60% of freshly isolated human peripheral γδ T cells, but downregulated after TCR signaling so that very few (< 10%) activated γδ T cells express CD28 [63–65]. CTLA-4 is expressed only marginally before and after PAg-stimulation on the cell surface [63]. Our data confirm the downregulation of CD28 in Vδ2^+^ T cells after TCR signaling, i.e., in this study after IPP/IL-15-stimulation, and also the only marginal expression of CTLA-4 before and after IPP/IL-15-stimulation (Supplementary Figure S9). In contrast, PD-1 and PD-L1 expression were significantly upregulated by the IPP-containing regimen. By using blocking antibodies, Peters et al. showed that CD28-CTLA-4/CD80-CD86 and PD-1-PD-L1 interactions are involved in the suppressive activity of Vδ2^+^ γδ T cells. Our experiments clearly confirm a role for the PD-1/PD-L1 axis. This finding also supports the notion that the TCR is decisive in inducing regulatory function in γδ T cells, as PD-L1 is positively regulated by TCR stimulation. We also demonstrate that CD28 stimulation on Vδ2^+^ T cells is not essential for Vδ2^+^ T cells to acquire suppressive function on autologous αβ T cells. However, since PD-1/PD-L1 interaction explains only about 50% of suppressive function of Vδ2^+^ T cells, CD28 stimulation on αβ T cells by suppressive Vδ2^+^ T cells is of relevance and needs to be elucidated as well as the role of other inhibitory receptors such as Tim-3, Lag-3 or TIGIT.

Similar to what is shown by Peters et al. [22], no correlation between Foxp3 expression and suppressive activity of Vδ2^+^ T cells was observed in the present study. On the contrary, the population with the least suppressive activity among PAg-stimulated Vδ2^+^ T cells (IPP/IL-15/IL-12 stimulation) had the highest Foxp3 expression and vice versa (IPP/IL-15 stimulation). This suggests that Foxp3 is a transient activation marker rather than a regulatory marker analogous to Foxp3 expression in activated human CD4 αβ T helper cells [66, 67]. Instead, as mentioned above we detected a significant correlation between PD-L1 protein expression and the suppressive activity of IPP-stimulated Vδ2^+^ T cells. This supports the finding that the interaction between PD-1 on αβ T cells and its ligand PD-L1 on γδ T cells restrains αβ T cell activation [19]. Interestingly, we demonstrate that PD-L1 expression is induced on γδ T cells by TCR-bypass stimulation as well as by PAgs [68] or by co-culture with αβ T cells as reported [22], but it is not significantly induced by a single strong TCR signal via TCR crosslinking (Fig. 2a bottom). Consequently, although our finding suggests that PD-L1 expression on Vδ2^+^ T cells can be a marker to predict their suppressive activity and respective blocking antibodies massively reduced inhibitory function, this prediction is not applicable to TCR-bypass-stimulated Vδ2^+^ T cells, which express high PD-L1 but exhibit no suppression, or positively freshly isolated Vδ2^+^ T cells, which have only marginal expression of PD-L1 but possess high suppressive activity. On the other hand, it is already known that activated γδ T cells express CD80/86 [49]. Although IPP-stimulated γδ T cells express CD80/86, which supports the priming and proliferation of αβ T cells, they also express PD-L1, the marker that can suppress αβ T cell proliferation. This, as well as inconsistency between PD-L1 expression and suppressive function, points to a potential regulatory mechanism hierarchically upstream of these molecules. Whether these regulatory mechanism(s) are on the effector cell or the target cell remains to be shown.

The finding that BTN3A1-exposed Vδ2^+^ T cells, like untouched and TCR-bypass-stimulated Vδ2^+^ T cells, did not exhibit any suppressive behavior, although BTN3A1 is suggested to be the natural Vδ2 TCR ligand, illuminates our incomplete understanding of what truly activates γδ T cells and those factors that contribute to Vδ2^+^ TCR signaling and thus (suppressive) effector differentiation in vivo. If assuming suppression resulting from crosslinking the TCR is 100%, IL-15 was able to significantly increase suppressive behavior further by 35% while other cytokines, such as TGF-β and IL-12, down-modulated inhibitory activity distinctively. This denotes a decisive role of environmental cues in (suppressive) effector differentiation of γδ T cells. The excellent performance of Vδ2^+^ T cells in terms of viability throughout the experimental procedures and the consistently distinct modulation of the TCR signal by cytokine stimulation strongly suggest that these TCR signals represent a physiological range of activation and function. Until we have identified the physiological (co-)ligand(s) and factors that contribute to Vδ2^+^ T-cell activation, we are unable to delineate what specifically promotes suppressive γδ T cells in vivo.

**Fig. 6:**
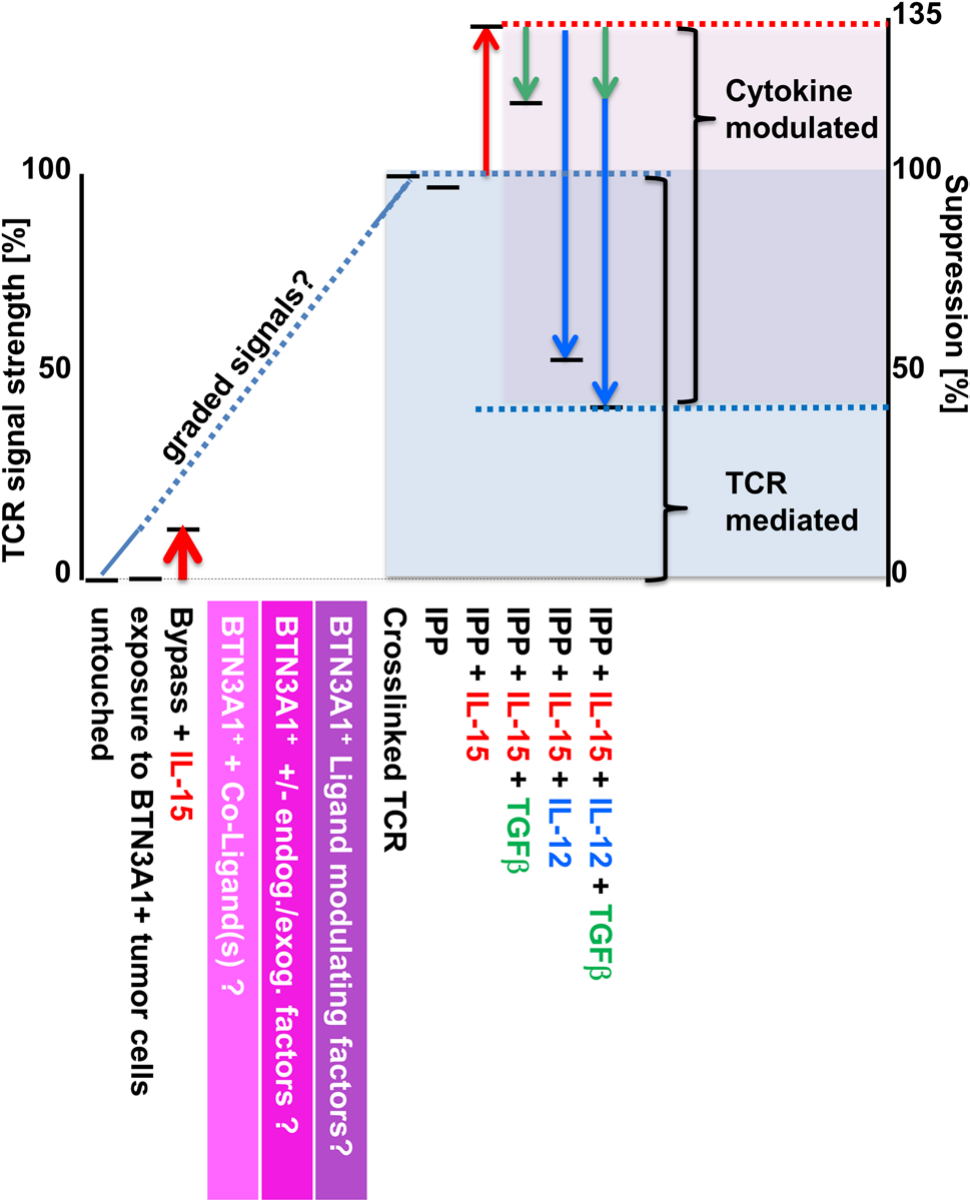
The suppressive activity of Vδ2^+^γδ T cells on αβ T cells is licensed by γδ TCR signaling and correlates with signal strength. When defining suppression resulting from TCR-crosslinking as 100%, environmental cues such as cytokines can modulate suppressive function further: While IL-15 (red arrows) increases suppressive activity of TCR-stimulated Vδ2^+^ T cells further, TGF-β (green arrows) and IL-12 (blue arrows) alone counter-regulate TCR/IL-15 stimulated Vδ2^+^ T cells suppressive behavior. TGF-β/IL-12 combined show additive effects in decreasing Vδ2^+^ suppressive function.

In summary, here we show for the first time that the suppressive activity of Vδ2^+^ T cells on αβ T cells is dependent on a TCR signal and correlates with its strength (Fig. 6). Accordingly, suppression correlates with and is dependent on a molecule that is also regulated via TCR stimulation: PD-L1. In the presence of a strong Vδ2^+^ TCR signal, functional maturation into a suppressive phenotype can be positively or negatively modulated by microenvironmental cues such as cytokines. Since direct contact with BTN3A1-expressing tumor cells alone does not bestow suppressive activity onto Vδ2^+^ T cells, further studies are needed to comprehensively identify what exactly activates Vδ2^+^ T cells and how activation translates into various specific effector functions. Thus, we first need to identify and understand its complete functional spectrum before we can utilize the full potential of the Vδ2^+^ effector T cell population in clinical settings.

### Electronic supplementary material

Below is the link to the electronic supplementary material.

Supplementary file1 (PDF 930 kb)

## Abbreviations

ANOVA: Analysis of variances
APC: Allophycocyanin
BFA: Brefeldin A
BTN: Butyrophilin
BTN3A1: Butyrophilin-3 isoform A1
CD: Cluster of differentiation
CTLA-4: Cytotoxic T-lymphocyte-associated protein 4
ER: Endoplasmatic reticulum
FACS: Fluorescence-activated cell sorting
FasL: Fas ligand
FITC: Fluorescein isothiocyanate
Foxp3: Forkhead box protein 3
GAPDH: Glycerinaldehyd-3-phosphate dehydrogenase
IL−: Interleukin−
IPP: Isopentenyl pyrophosphate
mAb: Monoclonal antibody
MLC: Mixed lymphocyte culture
PAgs: Phosphoantigens
PBMCs: Peripheral blood mononuclear cells
PCR: Polymerase chain reaction
PD-1: Programmed cell death protein 1
PD-L1: Programmed death ligand 1
PE: Phycoerythrin
RNA: Ribonucleic acid
RPMI: Roswell Park Memorial Institute
TCR: T cell receptor
TGF-ß: Transforming growth factor ß
TRAIL: TNF-related apoptosis-inducing ligand
TRAILR: TRAIL receptor
