# Supplementary material for: Suppressive activity of Vδ2+ γδ T cells on αβ T cells is licensed by TCR signaling and correlates with signal strength
Source: Cancer Immunol Immunother. 2020 Jan 25;69(4):593–610. doi: 10.1007/s00262-019-02469-8 (PMC7113223; doi:10.1007/s00262-019-02469-8)
Supplement: Supplementary file 1 — Supplementary file1 (PDF 930 kb) [file 262_2019_2469_MOESM1_ESM.pdf]

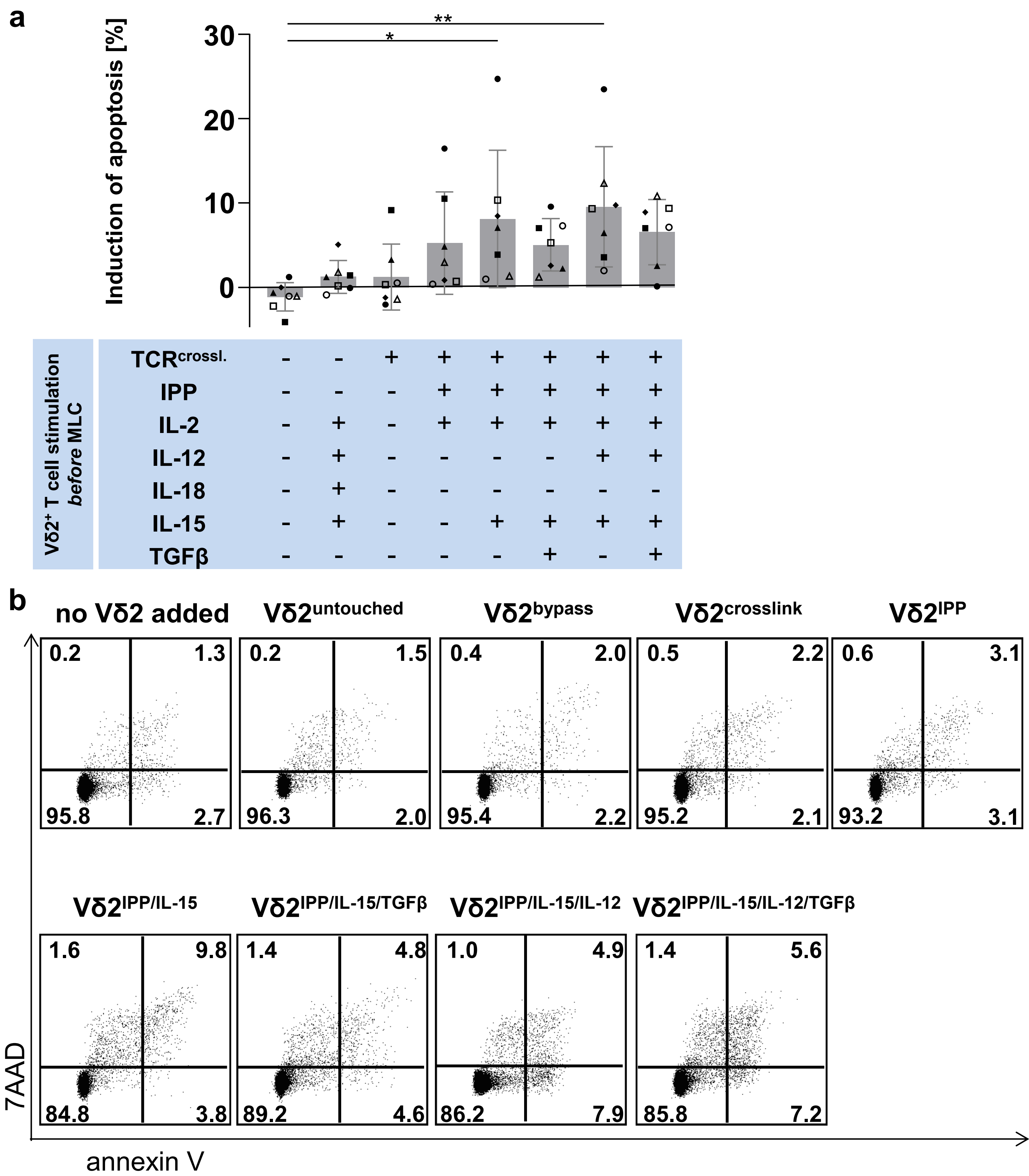

Supplementary Figure S1: Apoptosis induction of αβ T cells by autologous Vδ2^{+} T cells. Isolated Vδ2^{+} T cells after indicated stimulations were cultured at a 1:1 cell ratio with autologous PBMCs activated by anti-CD3/anti-CD28 Dynabeads. On day 1 of MLC, apoptotic αβ T cells were measured with flow cytometry and relative frequency of apoptotic cells, defined as annexin V^{+} cells, was calculated by subtracting the frequency of annexin V^{+} cells in CD3/CD28-stimulated αβ T cells in the absence of Vδ2^{+} T cells. (a) Each symbol indicates an individual donor culture. The data were generated using Vδ2^{+} T cells obtained from 7 different healthy donors. One-way ANOVA followed by Tukey's multiple comparison test was used. Bars represent the mean ± SD. * = $p < 0.05$, ** = $p < 0.01$, *** = $p < 0.001$, **** = $p < 0.0001$. Untouched: negatively isolated fresh Vδ2+ T cells. Crosslink: fresh positively isolated Vδ2^{+} T cells. (b) Flow cytometry plots of annexin V and 7AAD on αβ T cells from one representative donor are shown together with their corresponding negative control (without co-culture with Vδ2^{+} T cells).

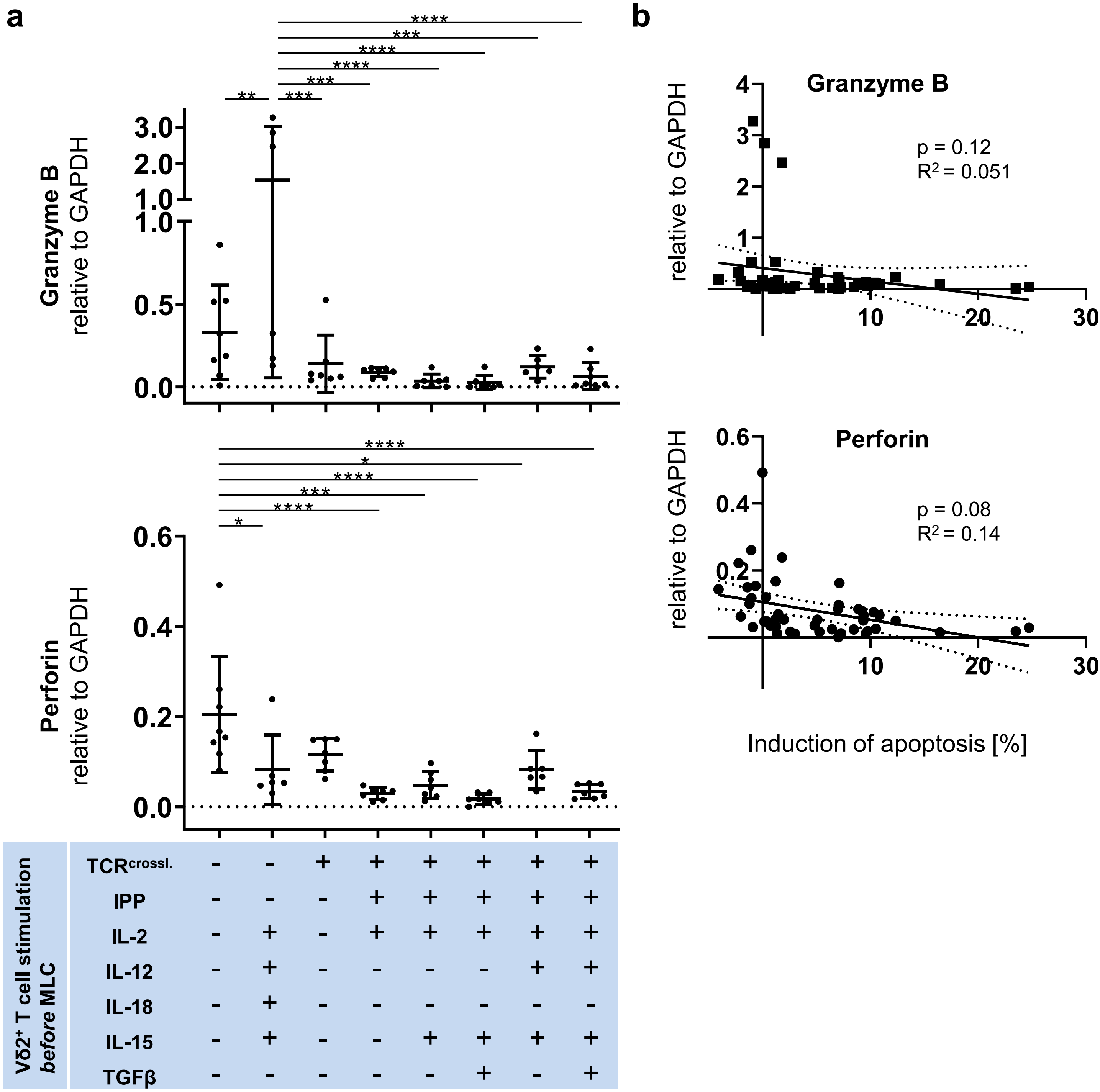

Supplementary Figure S2: (a) Expression of granzyme B and perforin in Vδ2+ T cells. Vδ2+ T cells were isolated and cultured as described in Materials and Methods. Vδ2+ T cells were analyzed by qPCR. The data were generated using Vδ2+ γδ T cells obtained from 7 different donors. One-way ANOVA followed by Tukey's multiple comparison test was used. Bars represent the mean ± SD. * = p <0.05, ** = p <0.01, *** = p <0.001, **** = p <0.0001. **(b) Correlation between mRNA levels of cytotoxic granules and apoptosis induction.** The relationships between granzyme B and perforin mRNA expression on Vδ2+ T cells and their apoptosis induction activity were compared using Fisher's exact test; the linear relationships were determined using Pearson's test.

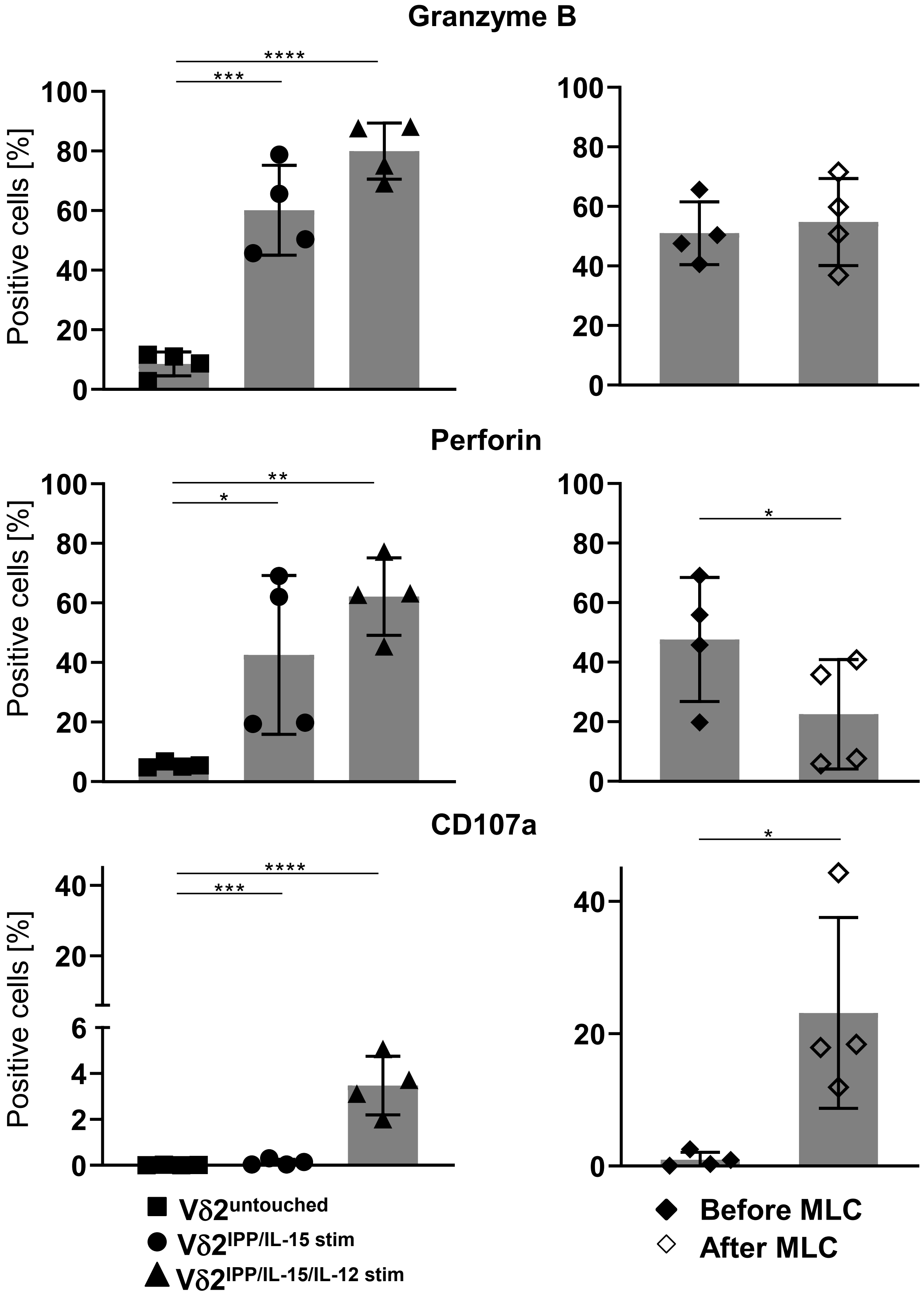

Supplementary Figure S3: CD107a, granzyme B and perforin expression in diversely stimulated Vδ2⁺ T cell populations. Vδ2⁺ T cells were isolated and cultured as described in Materials and Methods. Expression of CD107a, granzyme B and perforin in untouched, IPP/IL-15-stimulated, and IPP/IL-15/IL-12-stimulated Vδ2⁺ T cells were analyzed by flow cytometry (left). Expression of CD107a, granzyme B and perforin in IPP/IL-15-stimulated Vδ2⁺ T cells was analyzed before and after 4 hours of MLC (right). In MLC, isolated IPP/IL-15-stimulated Vδ2⁺ T cells were co-cultured with autologous PBMCs. FITC-anti-CD107a antibody was simultaneously added directly to the cell culture medium and cells were incubated for 4 hours at 37°C with 10 µg/ml of brefeldin A (Biolegend) as well as 6 µg/ml of monensin (Golgi-Stop, BD). Then granzyme B and perforin were intracellularly stained as described in Materials and Methods. Bars represent the mean ± SD. * = $p < 0.05$, ** = $p < 0.01$, *** = $p < 0.001$, **** = $p < 0.0001$.

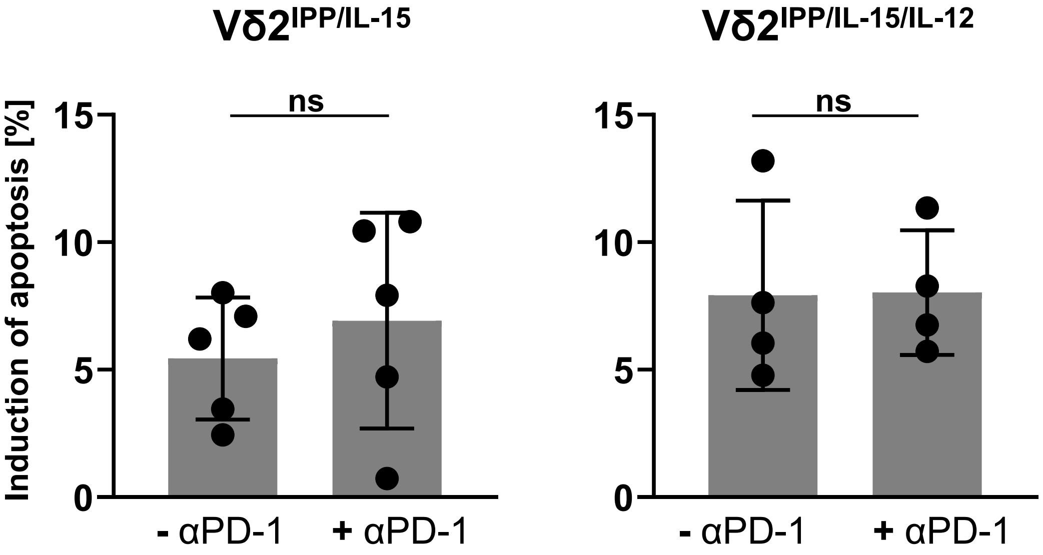

Supplementary Figure S4: Induction of apoptosis in αβ T cells in the presence of suppressive Vδ2⁺ T cells and an anti-PD-1 antibody (Pembrolizumab). Vδ2⁺ T cells were stimulated with IPP/IL-15 (n=5) or with IPP/IL-15/IL-12 (n=4) for the apoptosis assay as described in Materials and Methods. Positively isolated Vδ2⁺ T cells were cultured at a 1:1 cell ratio with autologous PBMCs activated by anti-CD3/anti-CD28 Dynabeads. PD-1/PD-L1 interaction in MLC was blocked by Pembrolizumab at a concentration of 5 µg/ml. On day 1 of MLC, apoptotic αβ T cells were measured with flow cytometry. Apoptotic cells were defined as annexin V⁺ cells and relative frequency of apoptotic cells was calculated by subtracting the frequency of annexin V⁺ cells in CD3/CD28-stimulated αβ T cells in the absence of Vδ2⁺ T cells. Student's t test was used to assess significance. Bars represent the mean ± SD. ns; not significant.

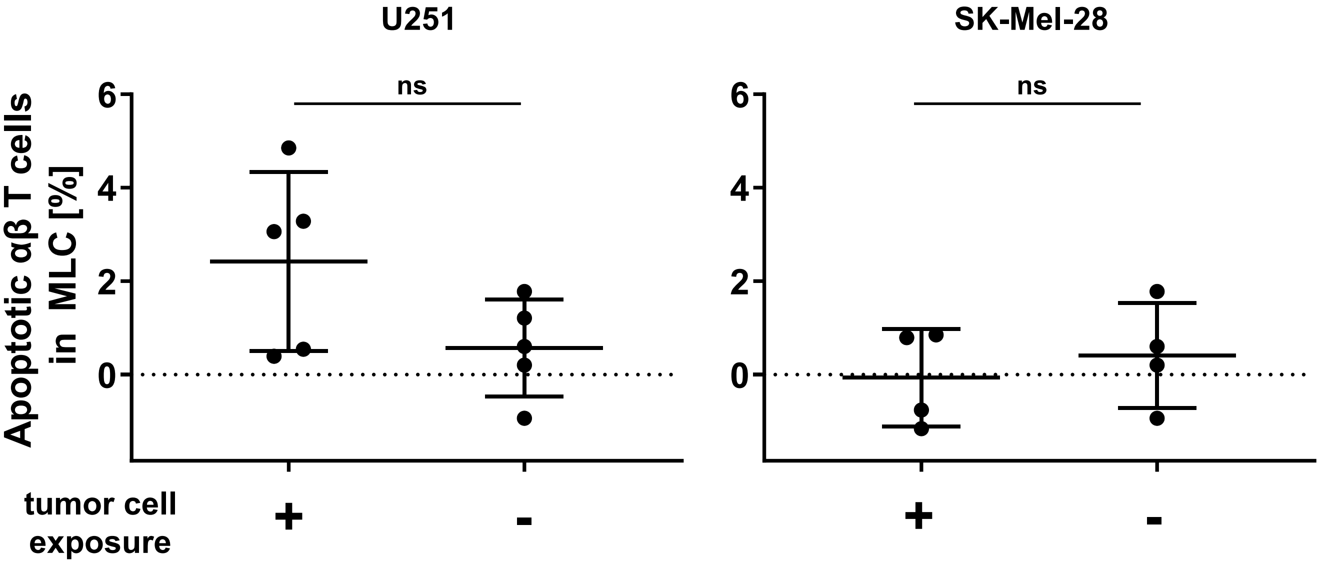

Supplementary Figure S5: BTN3A1-expressing tumor cells do not endow Vδ2$^+$ T cells with apoptosis inducing function. After 48-hour co-culture of untouched Vδ2$^+$ T cells with BTN3A1 expressing U251 or SK-Mel-28 cells, Vδ2$^+$ T cells were harvested and subsequently co-cultured at a 1:1 cell ratio with autologous PBMCs that had been activated by anti-CD3/anti-CD28 Dynabeads for 48 hours. On day 1 of MLC, apoptotic αβ T cells were measured with flow cytometry. Apoptotic cells were defined as annexin V$^+$ cells and relative frequency of apoptotic cells was calculated by subtracting the frequency of annexin V$^+$ cells in CD3/CD28-stimulated αβ T cells in the absence of Vδ2$^+$ T cells. The data were obtained using Vδ2$^+$ T cells from 5 different healthy donors for U251 and 4 different healthy donors for SK-Mel-28. Student's test was used to assess significance. Bars represent the mean ± SD. ns; not significant.

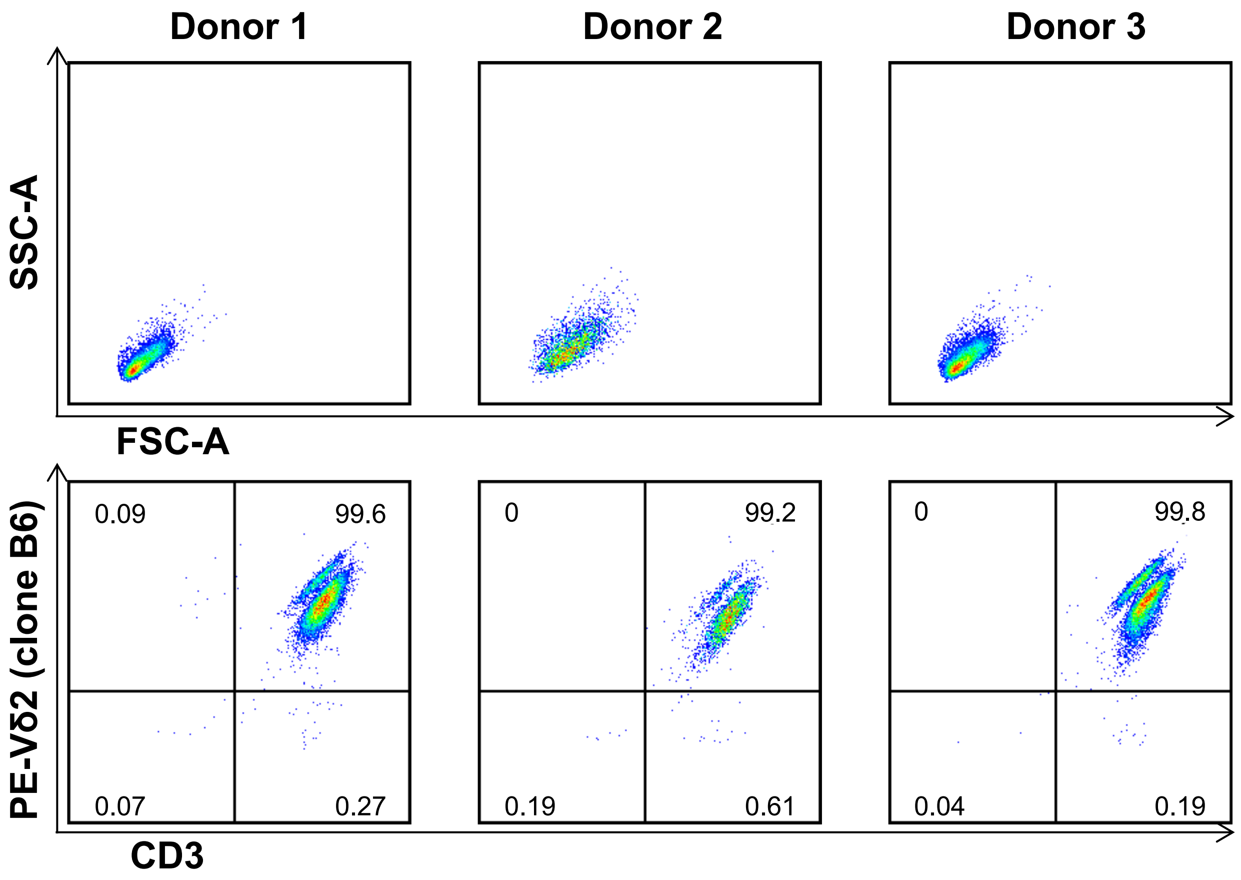

Supplementary Figure S6: Purity of Vδ2$^+$ T cells after positive (TCR crosslink) isolation used for the experiments shown in Figure 5a. Vδ2$^+$ T cells were positively isolated from PBMCs by using PE-anti-Vδ2 antibody (clone B6, Miltenyi Biotec) and anti-PE MicroBeads. Isolated cells were stained with the live/dead fixable violet dead cell stain kit (Invitrogen), PE-anti-Vδ2 antibody (clone B6, Miltenyi Biotec), and PerCP-anti-CD3 antibody (clone UCHT1, BioLegend). Shown are all live cells plotted for FSC-A/SSC-A (top) – to show that there are no contaminating myeloid cells in the isolates - and plotted for CD3/Vδ2 (bottom). Results from three representative donors are shown.

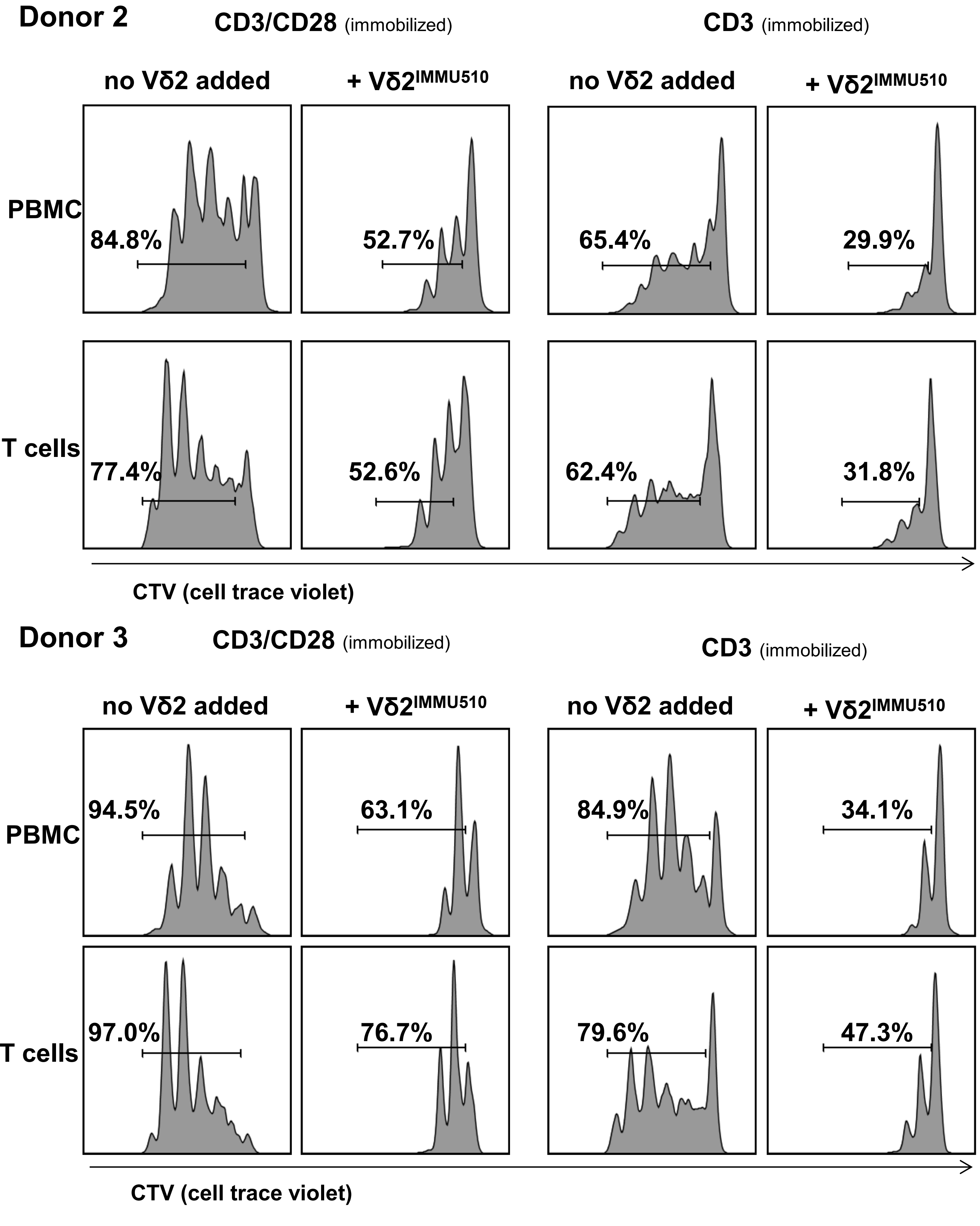

Supplementary Figure S7: CD28 stimulation is not essential for $V\delta2^+$ T cells to suppress autologous αβ T cells. Suppressive activity of $V\delta2^+$ T cells on autologous αβ T cells without CD28-stimulation. $V\delta2^+$ T cells were positively isolated from fresh peripheral blood and stimulated by immobilized anti-TCRγδ antibody (IMMU510, 1 μg/ml) for 24 hours and then co-cultured at a 1:1 cell ratio with autologous CTV-labelled autologous PBMCs or $CD3^+$ T cells on CD3- or CD3/CD28-coated wells. On day3 of MLC, proliferation of αβ T cells was measured with flow cytometry. Shown are the representative data from two donors, donor2 and donor3 (data for donor 1 are shown in Figure 5a).

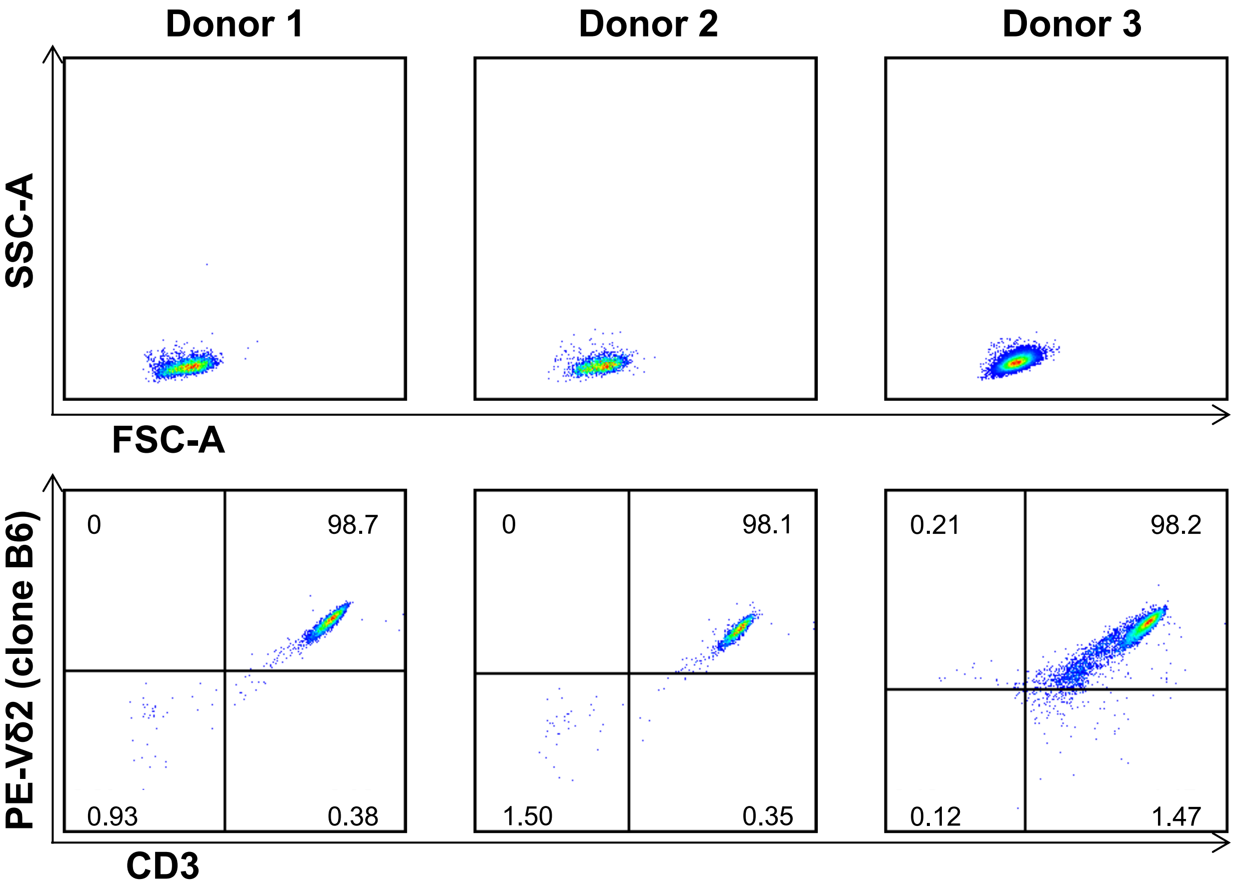

Supplementary Figure S8: Purity of Vδ2$^+$ T cells after negative (untouched) isolation used for the experiments shown in Figure 5b. Untouched Vδ2$^+$ T cells were isolated from freshly isolated PBMCs with a custom-made gammadelta T cell isolation kit (Stemcell Technologies) designed for negative isolation, and subsequently Vδ1$^+$ T cells were depleted *via* the combination of PE-anti-Vδ1 antibody (clone REA173, Miltenyi Biotec) and anti-PE MicroBeads. Aliquots of untouched isolated Vδ2$^+$ T cells were stained with the live/dead fixable violet dead cell staining kit (Invitrogen), FITC-anti-Vδ2 antibody (clone B6, BioLegend), and PerCP-anti-CD3 antibody (clone UCHT1, BioLegend). Shown are all live singlet cells plotted for FSC-A/SSC-A (top) and plotted for CD3/Vδ2 (bottom).

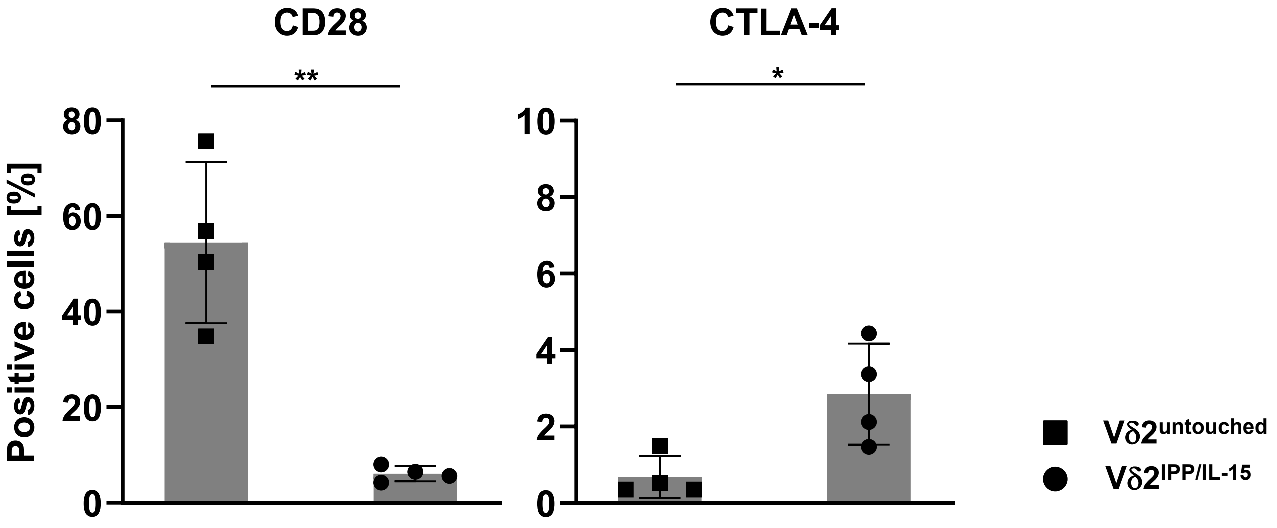

Supplementary Figure S9: Expression of CD28 and CTLA-4 in resting and activated Vδ2⁺ T cells. Vδ2⁺ T cells were isolated and cultured as described in Materials and Methods. Untouched and IPP/IL-15-stimulated Vδ2⁺ T cells were analyzed by flow cytometry for CD28 and CTLA-4 expression. The data were generated using Vδ2⁺ γδ T cells obtained from 4 different donors. Student's t test was used to assess significances. Bars represent the mean ± SD. * = p <0.05, ** = p <0.01.
